# Three-Class Correspondence Reliability for Deformation-Preserving ICP: Controlled Synthetic Evaluation and a Cross-Domain Stress Test

**DOI:** 10.3390/jimaging12100515

**Published:** 2026-10-15

**Authors:** Alpamis Kutlimuratov, Sultan Kudratov, Furkat Rakhmatov, Jamshid Khamzaev, Islambek Saymanov, Farkhod Qodirov, Sarbinaz Daniyarova, Hushnudbek Adinaev, Bekmurod Khujamuratov, Fazliddin Makhmudov

**Affiliations:** 1 Department of Applied Informatics, Kimyo International University in Tashkent, Tashkent 100121, Uzbekistan; kutlimuratov.aj@kiut.uz; 2 Department of Programming Technologies, Tashkent University of Information Technologies Named After Muhammad al-Khwarizmi, Tashkent 100121, Uzbekistan; s.kudratov@gmail.com (S.K.); f.raxmatov@tuit.uz (F.R.); 3 Department of Computer Systems, Tashkent University of Information Technologies Named After Muhammad al-Khwarizmi, Tashkent 100121, Uzbekistan; hamzayevjf@gmail.com; 4 School of Mathematics and Natural Sciences, New Uzbekistan University, Mustaqillik Ave. 54, Tashkent 100007, Uzbekistan; i.saymanov@newuu.uz; 5 Applied Mathematics and Intelligent Technologies Faculty, National University of Uzbekistan, Tashkent 100174, Uzbekistan; 6 Department of Econometrics, Tashkent State University of Economics, Tashkent 100066, Uzbekistan; f.qodirov@tsue.uz; 7 Department of Mathematical Analysis, Karakalpak State University, Nukus 230100, Uzbekistan; sdaniarova33@gmail.com; 8 Department of Computer Engineering, Urgench State University, Urgench 220100, Uzbekistan; hushnudbek.adinaev@gmail.com; 9 Department of IT Laboratory, Urgench RANCH University of Technology, Urgench 220511, Uzbekistan; bekmurod3388@gmail.com; 10 Department of Computer Engineering, Gachon University, Seongnam 13120, Republic of Korea; 11 Department of Digital Technology and Mathematics, Kokand University, Kokand 150700, Uzbekistan

**Keywords:** point-cloud registration, iterative closest point, deformation-aware registration, robust estimation, correspondence classification, synthetic-to-real domain shift, interpretable machine learning

## Abstract

Multi-epoch point-cloud analysis requires rigid alignment without allowing genuine geometric change to bias the estimated pose. This study presents a reliability-guided iterative closest point (ICP) formulation in which a compact classifier assigns each candidate correspondence to stable, deformation-candidate, or unreliable classes. Stable-class scores softly weight a Cauchy-robust point-to-plane objective on the special Euclidean group $SE\left(3\right)$, while deformation candidates are retained for signed-residual mapping. Controlled evaluation comprised 28 factor-level blocks, with 20 independently generated random trials per block and 560 paired trials per method. The three-class method achieved median rotational and translational errors of 0.227 degrees and 4.103 mm and 96.61% registration recall. Its principal benefit was changed-region discrimination: the F1 score (harmonic mean of precision and recall) reached 0.524 for the 5 and 10 mm cases, compared with 0.413 for binary classifier-guided ICP. However, the binary method produced slightly better rigid-registration accuracy, and the three-class stable-region false-positive rate was 6.71%. An external stress test on a Stanford Bunny scan pair used 60 controlled initialisations. Point-to-point ICP achieved 100% success, whereas the unretrained three-class method achieved 0% and assigned 39.3% of rigid-object correspondences to the deformation class. The results support three-way correspondence modelling as a controlled proof of concept but demonstrate that balanced synthetic training does not yield calibration-free transfer. Real-domain calibration, stronger geometric baselines, and validation with independent displacement ground truth are required before deployment.

## 1. Introduction

Repeated three-dimensional scanning is increasingly used to document the geometry of bridges, tunnels, retaining walls, façades, industrial facilities, and other engineering assets. Unlike sparse point sensors, a laser scanner records a dense spatial representation of visible surfaces and therefore supports area-wide inspection, geometric modelling, and comparison between monitoring epochs. Reviews and applied studies show that terrestrial laser scanning (TLS) can contribute to deformation measurement and structural assessment, while also showing that the final accuracy depends on acquisition geometry, registration, surface extraction, noise suppression, and the selected cloud-comparison method [1,2,3,4,5,6,7,8,9,10].

Multi-epoch deformation analysis requires the observations to be expressed in a common coordinate system. When external targets or a stable geodetic network are unavailable, registration is commonly performed from the measured geometry. ICP and its variants remain attractive because their objective functions are transparent and their outputs can be audited [11,12,13,14,15]. Nevertheless, nearest-neighbour correspondences and least-squares pose updates can fail under large initial misalignment, partial overlap, repetitive surfaces, local degeneracy, range noise, mixed pixels, and isolated or clustered outliers. Robust losses, correspondence trimming, covariance-aware distances, and globally oriented coarse-registration methods reduce some of these effects [14,15,16,17,18,19,20,21,22,23,24,25,26], but no single mechanism resolves all failure modes.

Structural monitoring introduces an additional distinction that is less central in conventional rigid-registration benchmarks. A large isolated residual is often a bad match, whereas a cluster of similarly directed residuals may represent bending, settlement, local bulging, joint movement, or another genuine physical change. If all large residuals are downweighted or if the registration is allowed to use the changed region as an anchor, the estimated rigid transformation can partially absorb the deformation. The resulting residual map may then underestimate the displacement that is of engineering interest. Conversely, preserving all large residuals can destabilize the pose estimate. The problem is therefore not merely to reject outliers, but to distinguish three roles: stable geometry that should constrain the global pose, coherent change that should be preserved for monitoring, and unreliable observations that should influence neither stage.

Learning-based registration has demonstrated that correspondence confidence, overlap information, and spatial consistency can improve matching under low overlap and high outlier ratios [27,28,29,30,31,32,33,34,35]. PREDATOR focuses attention on overlap regions [28], PointDSC embeds spatial consistency into learned outlier rejection [29], CoFiNet and GeoTransformer establish coarse-to-fine or geometry-aware correspondences [30,31], and RoITr improves rotation robustness [34]. These methods are valuable reference points, but end-to-end architectures can require large training sets, substantial computing resources, and domain-specific tuning. In engineering monitoring, available labelled scans are often limited and the final transformation must remain interpretable. Localizability-aware geometric methods such as X-ICP also demonstrate the value of explicitly diagnosing weak constraints rather than treating optimisation as a black box [36].

This study therefore adopts a hybrid design. A compact classifier does not regress the rigid pose directly; it produces correspondence-level class scores from interpretable geometric features. The pose is then estimated by robust point-to-plane optimisation with explicit weights, conditioning checks, and convergence criteria. The hypothesis tested here is deliberately limited: an explicit deformation class may improve changed-region discrimination under controlled corruption, while an external stress test can reveal whether the learned weighting transfers beyond its synthetic training domain. The study asks under which conditions the third class helps and records where it fails; it does not present an operational monitoring tool.

*The research addresses four questions:*

1. How does classifier-guided correspondence reliability affect rotational and translational accuracy relative to classical and robust ICP variants?
2. Does explicit modelling of a deformation class improve recovery of the imposed changed region relative to binary inlier/outlier classification?
3. How does the method respond to changes in noise, outlier ratio, overlap, density, initial misalignment, and deformation amplitude?
4. How do model complexity, runtime, unseen geometry, and an external real-scan domain affect the reliability of the learned weighting?

*The intended contributions are as follows:*

1. A correspondence-level three-class representation that separates stable anchors, coherent change candidates, and unreliable matches.
2. A deformation-preserving classifier-guided ICP objective in which the stable-class score controls a soft pose weight while change candidates remain available for residual mapping.
3. An interpretable robust optimisation on the special Euclidean group SE (3) that combines point-to-plane geometry, learned reliability scores, a Cauchy loss, and conditioning diagnostics.
4. A controlled synthetic protocol covering 28 factor-level blocks and reporting both rigid-registration and changed-region metrics.
5. An external real-scan stress test that documents a synthetic-to-real failure instead of assuming calibration-free transfer.

## 2. Related Work

### 2.1. Classical, Global, and Robust Registration

The original ICP formulation alternates correspondence search and rigid transformation estimation [11]. Point-to-plane minimisation can converge faster on locally smooth surfaces because it penalises displacement in the surface-normal direction [12,13]. Generalized ICP incorporates local covariance models and unifies point-to-point and point-to-plane interpretations [15]. Trimmed ICP addresses partial overlap by retaining a selected fraction of the lowest residuals [14]. Coherent point drift models one point set as Gaussian-mixture centroids and is useful when correspondence uncertainty is substantial [16].

ICP is a local optimiser. Go-ICP uses branch-and-bound search to provide a globally optimal rigid solution at higher computational cost [17], whereas fast global registration provides an efficient feature-based coarse stage [18]. Fast point feature histogram (FPFH) descriptors and random sample consensus (RANSAC) remain widely used for transparent initial alignment [19,20]. Robust statistics reduce the influence of large residuals through bounded or slowly growing losses [21,22,23]. Covariance analyses further show that registration uncertainty depends on geometry and correspondence structure, not only on the final root-mean-square error (RMSE) [24,25]. TEASER++ addresses extreme outlier contamination in putative correspondences using truncated least squares and certifiable estimation [26]. These methods provide strong baselines, but they generally classify a residual as geometrically consistent or inconsistent; they do not explicitly reserve a class for physically coherent structural change. Broader reviews of laser-scanning registration methods and large-scale TLS benchmarks are provided in [37,38].

### 2.2. Learning-Based Registration and Correspondence Rejection

Deep global registration predicts correspondence confidence and combines it with weighted geometric pose estimation [27]. PREDATOR learns overlap-aware features for low-overlap registration [28]. PointDSC uses spatial consistency during feature embedding and spectral matching [29]. CoFiNet extracts coarse-to-fine correspondences without a separate keypoint detector [30], while GeoTransformer encodes pairwise distances and triplet-wise angles in a geometry-aware transformer [31]. SC2-PCR develops second-order spatial compatibility for robust correspondence selection [32]. Lepard addresses partial matching in rigid and deformable scenes [33], RoITr introduces intrinsic rotation-invariant attention [34], and variational Bayesian correspondence rejection models ambiguity in long-range dependency learning [35].

These studies establish that learned confidence can be useful. The present method differs in purpose and scale. It does not seek to replace the complete registration pipeline or compete through network size. It asks whether a compact classifier can expose a monitoring-specific semantic distinction and guide an auditable ICP update. Logistic regression, gradient boosting, and a multilayer perceptron (MLP) are therefore evaluated under the same features. If a simpler classifier performs equivalently, the simpler option should be preferred. Recent reviews provide broader comparisons of classical and learning-based registration families [39,40].

### 2.3. Degeneracy, Uncertainty, and Localisability

A small residual does not guarantee a well-constrained transformation. Planar corridors, long cylinders, and repeated structural elements may leave one or more pose directions weakly observable. ICP covariance analyses quantify the dependence of pose uncertainty on surface geometry [24,25]. X-ICP performs correspondence-level localisability analysis and constrains updates in poorly observable directions [36]. The proposed work does not reproduce a full localisation framework, but it monitors the conditioning of the weighted normal matrix and reports failure when the stable correspondences do not provide sufficient constraints. Reliability classification and localisability are complementary: the first asks whether a correspondence should constrain the pose, while the second asks whether the retained constraints span the required degrees of freedom. In contrast to X-ICP, which diagnoses weak pose directions, the present classifier estimates correspondence role; the two mechanisms are therefore complementary rather than interchangeable.

### 2.4. Point-Cloud Deformation Monitoring

Point-cloud registration and change detection have long been linked in repeated laser-scanning analysis. Multiscale model-to-model cloud comparison (M3C2) estimates signed multiscale distances with local uncertainty and is widely used for surface change [1]. Repeated surface matching has been applied to land deformation [2], while structural studies show that registration, segmentation, reference-surface selection, and outlier treatment directly affect inferred displacement [3,4,5,6,7,8,9,10]. A recent perspective further demonstrates that the definition of correspondence determines whether an analysis estimates surface distance, displacement vectors, or parametric change [41].

Automatic selection of stable regions is therefore not a new problem. Wujanz et al. identified stable areas in unreferenced laser scans for deformation measurement [42]. Yang and Schwieger used supervoxel-based stable-area identification for targetless registration of deformed point clouds [43]. Piecewise-ICP subsequently selected stable planar patches, incorporated a level-of-detection threshold, and evaluated synthetic and real four-dimensional point clouds [44]. These studies directly connect registration with change-aware stable-area selection. Supervoxel stable-area identification and Piecewise-ICP operate at region or patch level, whereas the present formulation assigns point-level class scores and uses the stable score as a soft pose weight. Direct end-to-end numerical comparison with these methods remains necessary.

The narrower question addressed here is whether a compact, point-level three-class model can represent stable correspondences, coherent change candidates, and random outliers simultaneously and use the resulting stable-class score inside an auditable robust ICP update. This differs from patch-based stable-area selection but does not replace it. Because Piecewise-ICP and the earlier stable-area methods were not included as experimental baselines, the present results support only the tested proof of concept and not a state-of-the-art superiority claim.

## 3. Problem Formulation

Let the reference-epoch and current-epoch point clouds be

(1) $$P={\left\{p_{i}\in R^{3}\right\}}_{i=1}^{N},\;\;Q={\left\{q_{j}\in R^{3}\right\}}_{j=1}^{M}.$$

Here, N and M are the numbers of retained points after identical preprocessing. A current-epoch observation is modelled as

(2) $$q_{j}=R_{⋆}\left(x_{j}+u_{j}\right)+t_{⋆}+\varepsilon_{j}+o_{j}$$

where $x_{j}$ is the undeformed structural position, $u_{j}$ is a possible physical displacement, $R_{⋆}\in SO\left(3\right)$ and $t_{⋆}\in R^{3}$ represent scanner-pose change, $\varepsilon_{j}$ is measurement noise, and $o_{j}$ represents gross corruption or an outlier process. Occlusion and partial overlap are represented by points that have no valid counterpart in the other cloud.

The rigid transformation is

(3) $$T=\left[\begin{array}{cc} R & t\\ 0^{T} & 1 \end{array}\right]\in SE\left(3\right)$$

At iteration k, the nearest-neighbour index and the distance-gated candidate correspondence set are

(4) $$\begin{array}{ll} j_{i}^{\left(k\right)} & =\underset{l\in \{1,\ldots ,M\}}{arg\;min}{∥R_{k}p_{i}+t_{k}-q_{l}∥}_{2},\\ C^{\left(k\right)} & =\left\{\left(i,j_{i}^{\left(k\right)}\right):{∥R_{k}p_{i}+t_{k}-q_{j_{i}^{\left(k\right)}}∥}_{2}<d_{max}\right\}. \end{array}$$

Each correspondence has a latent class $c_{ij}\in \{S,D,O\}$: stable structural inlier (S), coherent deformation candidate (D), or outlier/unreliable match (O). Here, $d_{max}>0$ is the maximum admissible Euclidean distance for a candidate correspondence. The pose must be estimated primarily from class S. Class D must be retained for change analysis, and class O must be strongly downweighted.

## 4. Proposed Method

### 4.1. Preprocessing and Coarse Alignment

**Figure 1:**
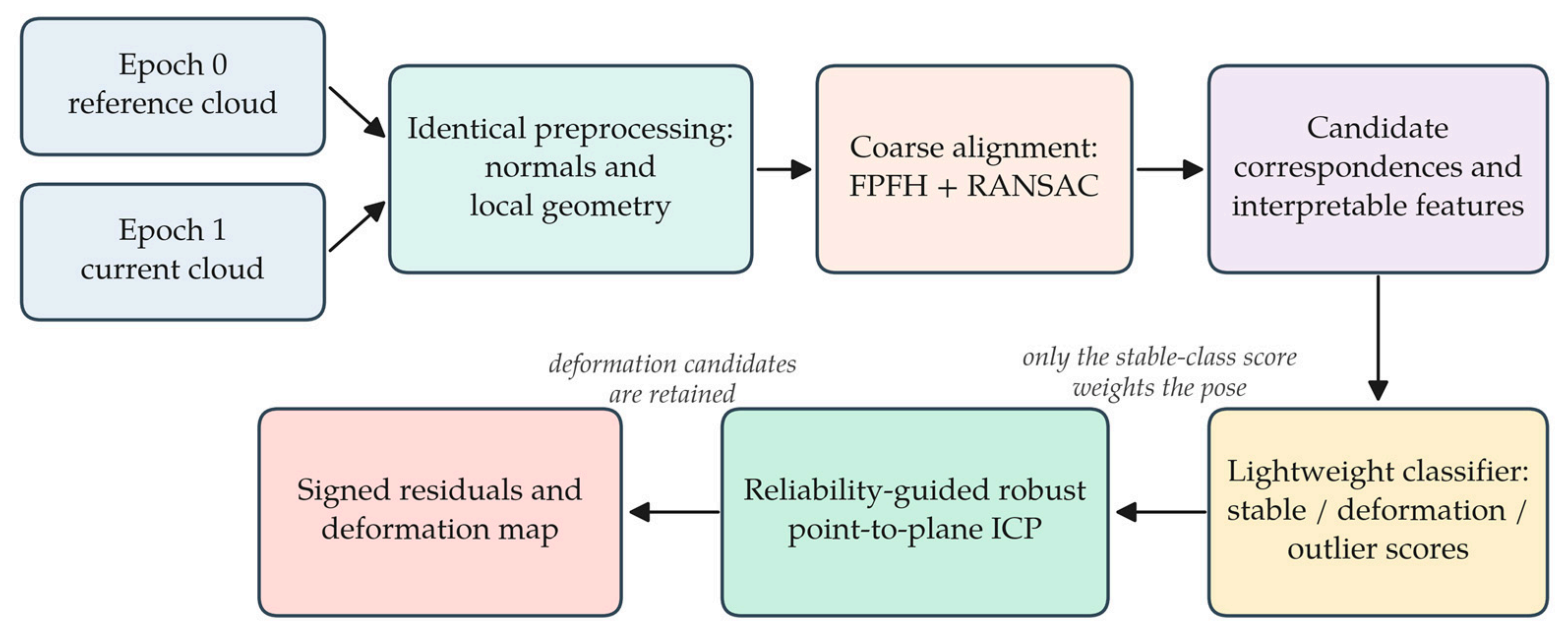
Complete pipeline from two monitoring epochs to reliability-guided robust ICP and a deformation map. Boxes follow the processing order; the two italic notes mark where the stable-class score enters the pose estimate and where deformation candidates are kept for mapping.

Figure 1 summarises the complete processing workflow. Invalid coordinates and scanner-specific no-return codes are removed. Optional voxel downsampling uses the same physical voxel size for all compared methods. Statistical or radius filtering is permitted only when it is applied identically to every baseline and its parameters are reported. Surface normals are estimated with either $k_{n}$ nearest neighbours or a physical radius $r_{n}$ selected relative to point spacing. The reference normal orientation convention is documented.

A coarse transform is obtained from known scanner poses or from FPFH correspondences, followed by RANSAC [18,19,20]. No method receives a better initial pose than another. The controlled experiments also bypass coarse registration and impose known rotation and translation errors, enabling the convergence basin to be measured directly.

### 4.2. Interpretable Correspondence Features

For, define the Euclidean and signed point-to-plane residuals [12,13] $\left(i,j\right)\in C^{\left(k\right)}$

(5) $$\begin{array}{cc} d_{ij}=∥Rp_{i}+t-q_{j}{∥}_{2}, & r_{ij}=n_{j}^{T}\left(Rp_{i}+t-q_{j}\right) \end{array}$$

Here, R and t are the current pose estimate, and $n_{j}$ is the unit normal at $q_{j}$. Let $\lambda_{1}\geq \lambda_{2}\geq \lambda_{3}\geq 0$ be the eigenvalues of the neighbourhood covariance matrix. The local linearity, planarity, curvature, and scattering measures are

(6) $$\begin{array}{llll} L & =\frac{\lambda_{1}-\lambda_{2}}{\lambda_{1}+\epsilon_{\lambda }}, & \Pi & =\frac{\lambda_{2}-\lambda_{3}}{\lambda_{1}+\epsilon_{\lambda }},\\ \kappa & =\frac{\lambda_{3}}{\lambda_{1}+\lambda_{2}+\lambda_{3}+\epsilon_{\lambda }}, & S_{c} & =\frac{\lambda_{3}}{\lambda_{1}+\epsilon_{\lambda }}. \end{array}$$

Here, $\epsilon_{\lambda }>0$ is a covariance-scale stabiliser with the same units as the eigenvalues $\lambda_{i}$.

For each candidate correspondence, the implemented 14-dimensional feature vector was

(7) $$f_{ij}={\left[\begin{array}{c} \frac{d_{ij}}{h},\;\frac{\left|r_{ij}\right|}{h},\;1-\left|n_{i}^{T}n_{j}\right|,\;\left|\Pi_{i}-\Pi_{j}\right|,\;max\left(\kappa_{i},\kappa_{j}\right),\\ \left|log\left(h_{i}/h_{j}\right)\right|,\;m_{ij},\;\frac{{med}_{N_{i}}\left(r\right)}{h},\;\frac{{MAD}_{N_{i}}\left(r\right)}{h},\;s_{i},\;v_{i},\\ 10^{3}\left|r_{ij}\right|,\;10^{3}\left|{med}_{N_{i}}\left(r\right)\right|,\;10^{3}{MAD}_{N_{i}}\left(r\right) \end{array}\right]}^{T}.$$

**Figure 2:**
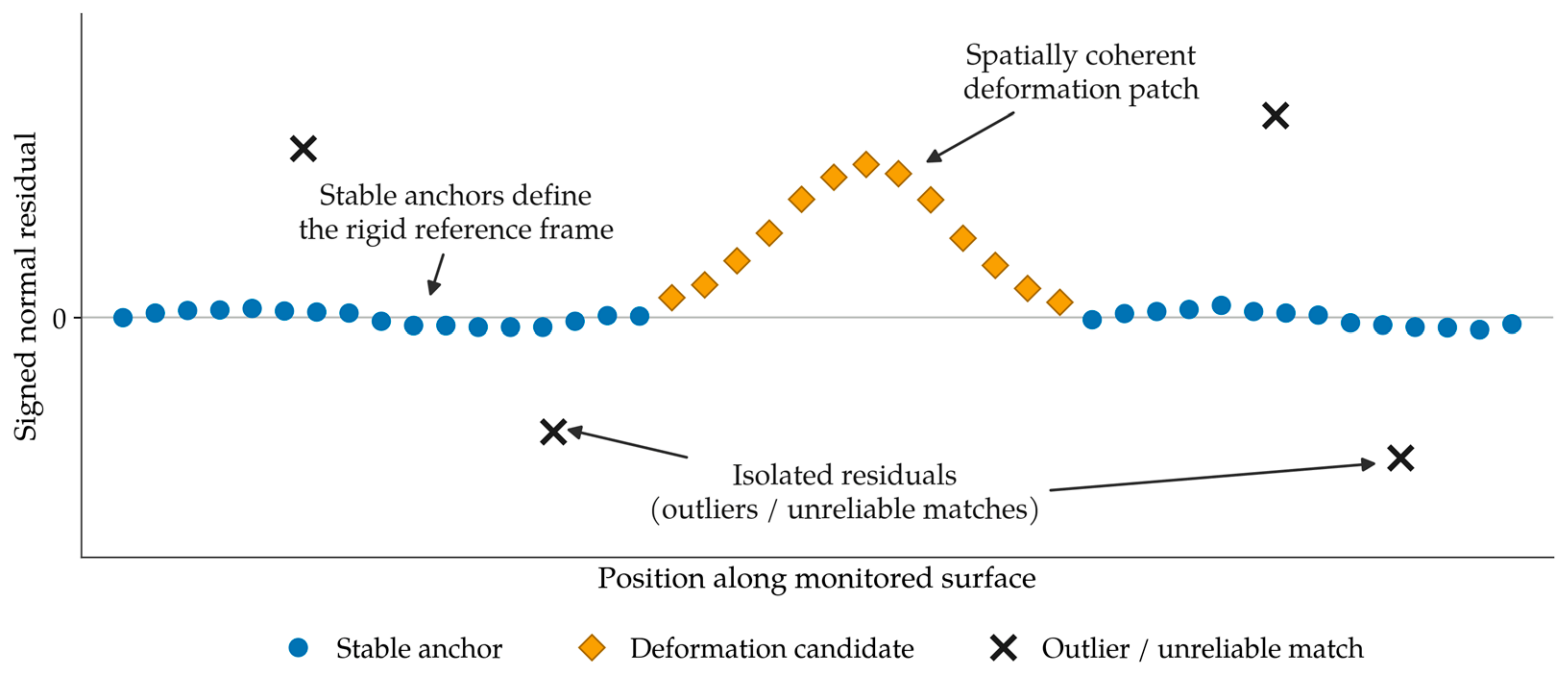
Physical interpretation of the three correspondence classes. Blue circular markers denote stable anchors, orange markers denote a spatially coherent deformation candidate, and the outlier/unreliable-match class is represented by an isolated residual conceptually distinct from the coherent patch. Arrows tie each annotation to the feature it describes; outliers are drawn as crosses.

Here, h is the nominal point spacing; $d_{ij}$ and $r_{ij}$ are the Euclidean and point-to-plane residuals for correspondence $\left(i,j\right)$, respectively; $n_{i}$ and $n_{j}$ are unit normals at $p_{i}$ and $q_{j}$, respectively; Π and κ denote planarity and curvature, respectively; $h_{i}/h_{j}$ is the local spacing ratio; $m_{ij}$ indicates mutual nearest-neighbour consistency; ${med}_{N_{i}}\left(r\right)$ and ${MAD}_{N_{i}}\left(r\right)$ denote the neighbourhood median residual and median absolute deviation, respectively; $s_{i}$ measures residual-sign incoherence; and $v_{i}$ is the fraction of valid residual neighbours. Coordinates and residuals are expressed in metres, so the last three components multiply residual quantities by $10^{3}$ to express them in millimetres. All features were clipped to finite ranges and standardised using training-set statistics only. Figure 2 illustrates the physical roles assigned to the three correspondence classes.

### 4.3. Lightweight Three-Class Classifier

The iterative reliability model was a compact multilayer perceptron (MLP) with standardised inputs, hidden widths 64–32–16, rectified linear unit (ReLU) activations, L2 regularisation α = 10^−4^, batch size 512, initial learning rate 10^−3^, and a three-unit softmax output. Training was limited to 100 epochs with early stopping after 12 validation epochs without improvement. The model remained sufficiently small for CPU inference.

(8) $$\pi_{ij}=softmax\;\left(g_{\theta }\left(f_{ij}\right)\right)={\left[\pi_{ij}^{S},\pi_{ij}^{D},\pi_{ij}^{O}\right]}^{T},\;\;\sum_{c\in \{S,D,O\}}\pi_{ij}^{c}=1$$

Here, $g_{\theta }$ denotes the learned multilayer-perceptron mapping parameterised by θ, and $\pi_{ij}^{c}$ is the score assigned to class c∈{S,D,O}.

Because the synthetic classes were balanced before model fitting to ensure adequate representation of all three classes, the training objective was ordinary multiclass cross-entropy,

(9) $$L=-\frac{1}{B}\sum_{i=1}^{B}\sum_{c\in \{S,D,O\}}y_{ic}logp_{ic}.$$

Here, B is the batch size; i indexes samples; c indexes classes; $y_{ic}$ is the one-hot target indicator; and $p_{ic}$ is the corresponding softmax output. No focal loss or explicit class weighting was used because the synthetic classes were balanced before fitting. Consequently, the outputs reflect the balanced training prior and were not calibrated to natural real-scan class prevalence. They are used below as relative reliability scores; calibrated deployment probabilities are not claimed. This balanced sampling changes the effective class prior; therefore, the softmax outputs are treated as relative reliability scores rather than calibrated probabilities under natural real-scan class prevalence.

Training labels were obtained from the known synthetic generation process. Stable labels represented valid correspondences outside the imposed deformation core; deformation labels represented points whose imposed normal displacement was at least 50% of the peak amplitude; and outlier labels covered injected points, nonoverlapping regions, and geometrically inconsistent correspondences. The 50% threshold is an operational labelling convention that identifies the central, high-amplitude portion of the imposed synthetic change; it is not an engineering damage threshold. The corner-frame geometry was never used during model fitting or threshold selection.

### 4.4. Reliability-Guided Robust Point-to-Plane ICP

The stable-class score becomes a soft pose weight,

(10) $$a_{ij}=clip\;\left({\left(\pi_{ij}^{S}\right)}^{\gamma },a_{min},1\right)$$

Here, γ>0 controls confidence sharpness, and $0<a_{min}\leq 1$ is a positive lower bound that prevents classifier-derived weights from vanishing completely during early iterations. The deformation-class score is not used as a negative pose weight; it is reserved for change analysis. The pose objective is

(11) $$\underset{R,t}{min}E\left(R,t\right)=\sum_{\left(i,j\right)\in C^{\left(k\right)}}a_{ij}\;\rho \;\left(\frac{r_{ij}}{\sigma_{r}}\right).$$

Here, $\sigma_{r}=1.4826\;MAD\left(\left\{r_{ij}:\left(i,j\right)\in C^{\left(k\right)}\right\}\right)+\epsilon_{r}$ is a positive robust residual scale computed over the current candidate set, and $\epsilon_{r}>0$ is a residual-scale floor with the same units as $r_{ij}$. The Cauchy loss is selected because it smoothly suppresses extreme residuals without a discontinuous rejection boundary:

(12) $$\rho \left(s\right)=\frac{c^{2}}{2}log\;\left(1+{\left(\frac{s}{c}\right)}^{2}\right),\;\;w_{\rho }\left(s\right)=\frac{1}{1+{\left(s/c\right)}^{2}}$$

Here, c>0 is the dimensionless Cauchy tuning constant. Because the normal equations are written for the unnormalised residual $r_{ij}$, the effective iteratively reweighted least-squares (IRLS) weight includes the scale factor $\sigma_{r}^{-2}$:

(13) $$w_{ij}=\frac{a_{ij}}{\sigma_{r}^{2}}\;w_{\rho }\;\left(\frac{r_{ij}}{\sigma_{r}}\right).$$

An incremental pose $\delta \xi ={\left[\delta \omega^{T},\delta v^{T}\right]}^{T}\in R^{6}$ is computed in the Lie algebra $se\left(3\right)$ using the left-multiplicative convention $T_{k+1}=exp\left(\delta \xi^{\wedge }\right)T_{k}$, where ${\left(\cdot \right)}^{\wedge }$ is the standard map from $R^{6}$ to $se\left(3\right)$. Linearising each residual under this convention gives

(14) $$r_{ij}\left(\delta \xi \right)\approx r_{ij}+J_{ij}\delta \xi ,\;\;J_{ij}=\left[{\left(\left(Rp_{i}+t\right)\times n_{j}\right)}^{T}\;\;n_{j}^{T}\right]$$

The weighted normal equations are

(15) $$\left(J^{T}WJ+\lambda I\right)\delta \xi =-J^{T}Wr.$$

Here, J stacks the Jacobians row $J_{ij}$, $W=diag\left(w_{ij}\right)$, and r stacks the residuals $r_{ij}$. The coefficient λ≥0 is a damping term activated only when conditioning requires it, and $I\in R^{6\times 6}$ is the identity matrix. The update is

(16) $$T_{k+1}=exp\;\left(\delta \xi^{\wedge }\right)T_{k}$$

Convergence requires both a small pose increment and a small objective decrease:

(17) $$\begin{array}{ll} ∥\delta \omega {∥}_{2} & <\tau_{R},\;\;∥\delta v{∥}_{2}<\tau_{t},\\ \frac{\left|E_{k+1}-E_{k}\right|}{E_{k}+\epsilon_{E}} & <\tau_{E}. \end{array}$$

Here, $\tau_{R}>0$, $\tau_{t}>0$, and $\tau_{E}>0$ are the rotation-increment, translation-increment, and relative-objective tolerances, respectively, and $\epsilon_{E}>0$ is an objective-scale floor that prevents division by zero. The condition number and eigenvalues of $J^{T}WJ$ are logged. If the stable correspondences fail to constrain the pose, the pair is declared degenerate rather than forced to converge.

### 4.5. Deformation-Preserving Post-Registration Analysis

After final registration, correspondences are recomputed without deleting points classified as deformation candidates. Target normals follow the orientation convention defined in Section 4.1; consequently, positive $\delta_{i}$ means that the transformed reference point lies in the $+n_{j_{i}}$ direction from the matched current-epoch point. Signed normal displacement is

(18) $$\delta_{i}=n_{j_{i}}^{T}\left(Rp_{i}+t-q_{j_{i}}\right)$$

A dimensionless deformation score is

(19) $$D_{i}=\pi_{i,j_{i}}^{D}\;\frac{\left|\delta_{i}\right|}{max\;\left(1.4826\;MAD\;\left(\left\{\delta_{l}:l\in N_{i}\right\}\right),\epsilon_{\delta }\right)}.$$

Here, $N_{i}$ is a local neighbourhood and $\epsilon_{\delta }>0$ is a displacement-scale floor, expressed in the same units as $\delta_{i}$, that prevents division by a vanishing local MAD. Candidate regions are formed using spatial connected components or density-based clustering and a minimum physical area or point count. This stage indicates a coherent change signal; it does not by itself diagnose damage or establish structural safety.

### 4.6. Computational Complexity

The complete registration procedure is summarised in Algorithm 1; the cost of its main steps is analysed below. Nearest-neighbour search with a k-dimensional (k-d) tree is approximately $O\left(NlogM\right)$ per iteration. Local descriptors are precomputed for both clouds. The MLP cost is $O\left(\left|C\right|H\right)$, where H is the small total hidden width, and is typically lower than correspondence search. Solving the six-parameter normal equations is constant in state dimension, although accumulation is $O\left(\left|C\right|\right)$. Runtime reporting separates preprocessing, coarse alignment, feature extraction, classifier inference, and ICP optimisation.
**Algorithm 1.** Deformation-preserving classifier-guided ICPInput: reference cloud P; current cloud Q; initial transform $T^{\left(0\right)}$; trained classifier $g_{\theta }$; maximum iterations K. Output: rigid transform $\hat{T}$; class scores; signed-residual and deformation-score maps.
Remove invalid points and apply identical optional filtering/downsampling.Estimate normals, neighbourhood covariance eigenvalues, and local densities.Obtain a transparent coarse alignment or apply the controlled initial pose.For k=0,…,K−1:Transform P by $T^{\left(k\right)}$.Construct candidate correspondences using a physical distance threshold.Compute interpretable feature vectors $f_{ij}$.Predict $\pi_{ij}^{S}$, $\pi_{ij}^{D}$, and $\pi_{ij}^{O}$.Compute stable reliability and robust IRLS weights.Assemble and solve the weighted point-to-plane normal equations.Update the pose on $SE\left(3\right)$ and evaluate conditioning.Stop when pose and objective convergence criteria are satisfied. 5.Recompute final correspondences and class scores.6.Calculate signed normal residuals and deformation scores.Apply spatial filtering only to the deformation map, not to the pose estimate.Return $\hat{T}$ and monitoring outputs.

## 5. Experimental Design and Data

### 5.1. Datasets and Evaluation Roles

The evaluation contains two completed blocks with distinct evidential roles. Block A uses a ribbed panel, cylindrical shell segment, and rectangular box beam for model development and controlled testing, while a corner-frame surface is withheld for geometry-generalisation testing. Block B uses a compact Stanford Bunny pair referenced by the Point Cloud Library regression tests. Block A evaluates recovery of analytically imposed deformation; Block B evaluates external rigid registration and synthetic-to-real transfer only. No experiment with independently measured physical deformation is included, so metrological or deployment claims are outside the scope of this study. Table 1 summarises the dataset roles and ground-truth status.

### 5.2. Synthetic Corruption and Deformation Fields

Four engineering-oriented parametric geometries were considered: a ribbed panel, a cylindrical shell segment, a rectangular box beam, and a corner frame. The first three geometries were used for model development and controlled testing, whereas the corner frame was withheld as an unseen geometry. The random seed was fixed at 20,260,716 for reproducibility.

For each clean reference point, the current-epoch observation was generated as

(20) $$q_{i}=R_{gt}\left(p_{i}+d_{i}\right)+t_{gt}+\varepsilon_{i}$$

where $p_{i}$ is the clean reference point; $d_{i}$ is the imposed physical displacement; $R_{\mathrm{gt}}$ and $t_{\mathrm{gt}}$ define the known rigid transformation between scanner frames; and $\varepsilon_{i}$ is zero-mean Gaussian coordinate noise. Isolated and clustered outliers were sampled independently, overlap was controlled by spatial cropping, and density variation was created by nonuniform subsampling.

The coherent deformation field was prescribed in the local surface-normal direction as

(21) $$d_{i}=Aexp\;\left[-\frac{1}{2}\left({\left(\frac{x_{i}-c_{x}}{s_{x}}\right)}^{2}+{\left(\frac{y_{i}-c_{y}}{s_{y}}\right)}^{2}\right)\right]n_{i}$$

**Figure 3:**
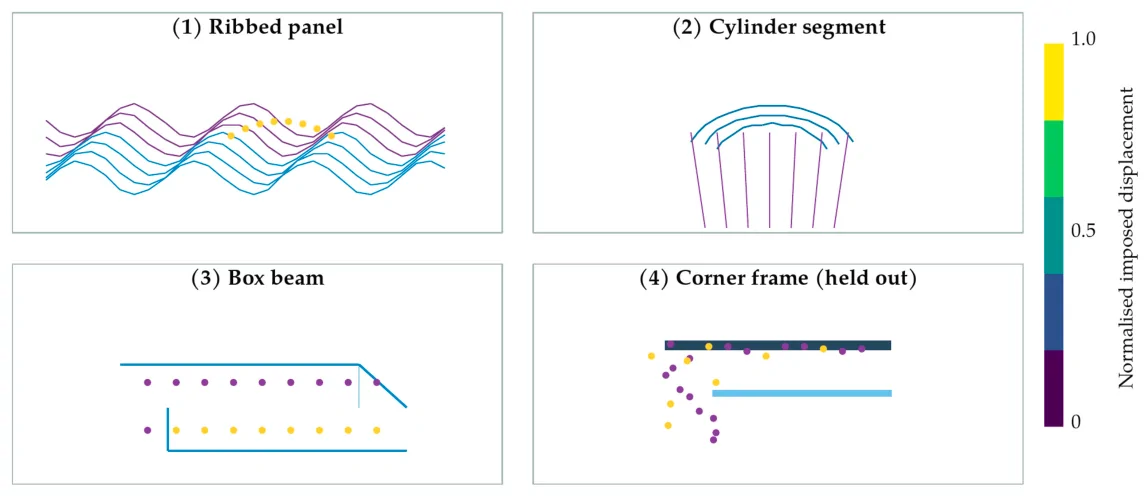
Synthetic engineering-oriented geometries: (**1**) ribbed panel, (**2**) cylinder segment, (**3**) box beam, and (**4**) held-out corner frame. The colour scale shows normalised imposed displacement; the corner frame was excluded from classifier training.

Here, A is the peak displacement amplitude; $\left(c_{x},\;c_{y}\right)$ is the centre of the changed region; $s_{x}$ and $s_{y}$ control its spatial extent; and $n_{i}$ is the local unit normal. The deformation class was defined as the core where the imposed displacement reached at least 50% of A. These fields provide controlled labels and are not claimed to reproduce every structural mechanism. Figure 3 visualises the four synthetic geometries and their imposed displacement patterns.

### 5.3. Data Splitting and Training

The correspondence dataset contained 12,675 balanced examples described by 14 physically interpretable features. A total of 9506 samples was used for training, 1267 for validation, and 1902 for internal testing. The split was performed at the generated-pair level, and the complete corner-frame geometry was reserved for external unseen-geometry evaluation.

The MLP used ReLU activations, L2 coefficient 10^−4^, batch size 512, initial learning rate 10^−3^, a maximum of 100 epochs, and early stopping. Logistic regression and histogram gradient boosting used the same feature vectors and splits. On the internal test subset, three-class macro-F1 values were 0.558 for logistic regression, 0.580 for gradient boosting, and 0.544 for the MLP.

Gradient boosting achieved the highest internal macro-F1, whereas the MLP was retained inside iterative ICP because its median inference time was 0.506 ms per 1000 correspondences and its serialised size was 94.0 kB, compared with 21.009 ms and 1065.5 kB for gradient boosting. This is an implementation trade-off rather than evidence that the MLP is statistically or end-to-end superior. A complete classifier selection would require all candidate models to be evaluated inside the same ICP loop.

### 5.4. Controlled Factors

A one-factor-at-a-time protocol was used to avoid an unnecessarily large full-factorial experiment. The study comprised 28 factor-level blocks and 20 independently generated random trials per block, giving 560 paired trials per method. The 28 blocks are the sum of the evaluated levels across six separate factor sweeps: Gaussian noise (5 levels), outlier ratio (5), overlap (4), initial rotation (5), retained density (4), and peak coherent deformation (5). In each sweep, the remaining factors were held at their reference settings. Nominally identical reference settings occurring in different sweeps were retained as separate, independently generated blocks; therefore, 28 denotes factor-level blocks rather than 28 distinct parameter vectors.

The evaluated levels were as follows: noise, 0, 1, 2, 5, and 10 mm; outliers, 0%, 10%, 20%, 30%, and 40%; overlap, 30%, 50%, 70%, and 90%; initial rotation, 0°, 5°, 10°, 20°, and 30°; retained density, 100%, 50%, 25%, and 12.5%; and peak deformation, 0, 1, 2, 5, and 10 mm. These six level sets contain 5 + 5 + 4 + 5 + 4 + 5 = 28 factor-level blocks. Table 2 summarises the six controlled factor sweeps.

### 5.5. Baselines and Fairness

Six registration variants were evaluated on identical synthetic pairs and initial transformations: point-to-point ICP, point-to-plane ICP, trimmed ICP, Cauchy-robust ICP without learned weights, binary classifier-guided ICP, and the proposed three-class classifier-guided ICP. The same candidate construction, distance limits, iteration budgets, and success criteria were used within the implemented framework. Generalized ICP (GICP), the stable-area methods [42,43], Piecewise-ICP [44], and external deep registration systems were not implemented. This still holds for the controlled synthetic benchmark. Simplified re-implementations of X-ICP [36], the supervoxel-based method [43] and Piecewise-ICP [44] were evaluated on public benchmarks (Section 6.7). Accordingly, the experiments compare the proposed formulation only with the listed baselines and do not establish state-of-the-art superiority.

Fast global registration (FGR) and FPFH matching with RANSAC are global coarse-alignment methods. They estimate the initial pose, which the controlled experiments impose directly (Section 4.1), so they are not alternatives to the local refinement stage compared here and were not run as baselines. Among the six variants, trimmed ICP, Cauchy-robust ICP and binary classifier-guided ICP are the robust references; point-to-point and point-to-plane ICP serve as lower bounds.

The complete fixed parameter configuration (normal-neighbourhood definitions, correspondence gates, robust-loss and convergence settings, trim fraction, and feature-clipping ranges) was held constant across all applicable comparisons and is available with the code from the corresponding author upon reasonable request.

### 5.6. Metrics and Statistical Analysis

For ground-truth rotation $R_{gt}$ and translation $t_{gt}$, the relative rotation error (RRE) and relative translation error (RTE) were computed as

(22) $$\begin{array}{ll} RRE & =\frac{180}{\pi }{cos}^{-1}\;\left[clip\;\left(\frac{tr\left(R_{gt}^{T}R_{est}\right)-1}{2},-1,1\right)\right],\\ RTE & =∥t_{est}-t_{gt}{∥}_{2}. \end{array}$$

Here, $R_{est}$ and $t_{est}$ are the estimated rotation and translation, respectively, and $clip\left(x,-1,1\right)$ clamps x to $\left[-1,1\right]$ before the inverse cosine. Registration recall was the proportion of trials satisfying $RRE<2^{\circ }$ and RTE<15 mm. Residual root-mean-square error (RMSE) was retained as a geometric diagnostic but was not treated as sufficient evidence when deformation was present. Change-analysis metrics included normal-displacement mean absolute error (MAE) and RMSE, peak-amplitude bias, changed-region F1, and the false-positive rate in known stable regions. In Table 3, numerical failure denotes solver breakdown or non-convergence and is distinct from a threshold failure counted through registration recall.

F1 denotes the harmonic mean of precision *P* and recall *R*, F1 = 2*PR*/(*P* + *R*), computed for the detected changed region against the imposed deformation core, and FPR denotes the false-positive rate in regions known to be stable. Macro-F1 is the unweighted mean of the per-class F1 values.

Because the same 560 synthetic pairs were processed by every algorithm, overall method effects were tested with the Friedman test followed by paired Wilcoxon signed-rank tests with Holm correction. Medians are emphasised because the error distributions were strongly skewed. The reported *p*-values should be interpreted together with the paired median differences and the practical registration thresholds rather than as stand-alone evidence of engineering relevance.

### 5.7. External Real-Scan Stress Test and Cross-Domain Protocol

The external real-scan experiment used the Stanford Bunny pair bun0.pcd and bun4.pcd referenced by the Point Cloud Library registration regression tests [45,46]. The underlying Stanford Bunny range scans were acquired with a Cyberware 3030 MS scanner and are part of the Stanford 3D Scanning Repository [47,48]. The source and target fragments contained 397 and 361 points, respectively. Their median local spacing was 7.997 mm, and the estimated symmetric overlap was 96.2% using an 11.995 mm proximity threshold. This single compact rigid pair is used only as an external stress test and is not sufficient to establish general real-world transfer or deformation-monitoring validity.

The numerical transformation embedded in the PCL regression test was used as the scoring reference. Its rotation block was projected to the nearest proper rotation matrix by singular-value decomposition before RRE calculation. This transform is a software regression reference rather than traceable metrological ground truth; consequently, Block B evaluates external rigid-registration transfer only and does not validate physical deformation amplitude.

Two protocols were evaluated. First, all methods started from the identity transform; relative to the numerical reference, this represented an initial rotation error of 28.323° and a translation error of 53.747 mm. Second, 60 controlled starts were generated from five rotation levels (0°, 5°, 10°, 15°, and 20°) and four translation levels (0, 5, 10, and 20 mm), with three random directions per combination and random seed 20,260,717. The same six registration variants used in Block A were applied. The binary and three-class classifiers were transferred without retraining. Because the object is compact and the numerical reference is precise, success required RRE < 2° and RTE < 5 mm.

## 6. Results

### 6.1. Overall Registration Accuracy

Across 560 controlled trials per method, the proposed three-class classifier-guided ICP achieved a median RRE of 0.227° and a median RTE of 4.103 mm; registration recall was 96.61%. It substantially outperformed ordinary point-to-point, point-to-plane, and Cauchy-robust ICP. However, binary classifier-guided ICP produced a slightly lower median RTE of 3.549 mm and a higher recall of 97.86%. The proposed method therefore did not provide universal superiority in rigid-registration accuracy; its main benefit was associated with deformation-aware monitoring.

The Friedman test detected an overall method effect for RTE (χ^2^ = 1497.972, *p* < 10^−16^). Relative to the three-class method, median RTE was higher by 5.328 mm for point-to-point ICP (95% CI 4.704–6.220 mm), 24.871 mm for point-to-plane ICP (21.425–27.287 mm), and 4.547 mm for Cauchy-robust ICP (3.799–5.299 mm), with Holm-corrected *p* < 10^−67^. Trimmed ICP differed by only 0.090 mm (95% CI −0.092 to 0.334 mm), whereas binary classifier-guided ICP was better by 0.226 mm (Holm-corrected *p* = 8.47 × 10^−9^).

### 6.2. Sensitivity to Noise, Overlap, Density, and Initial Pose

**Figure 4:**
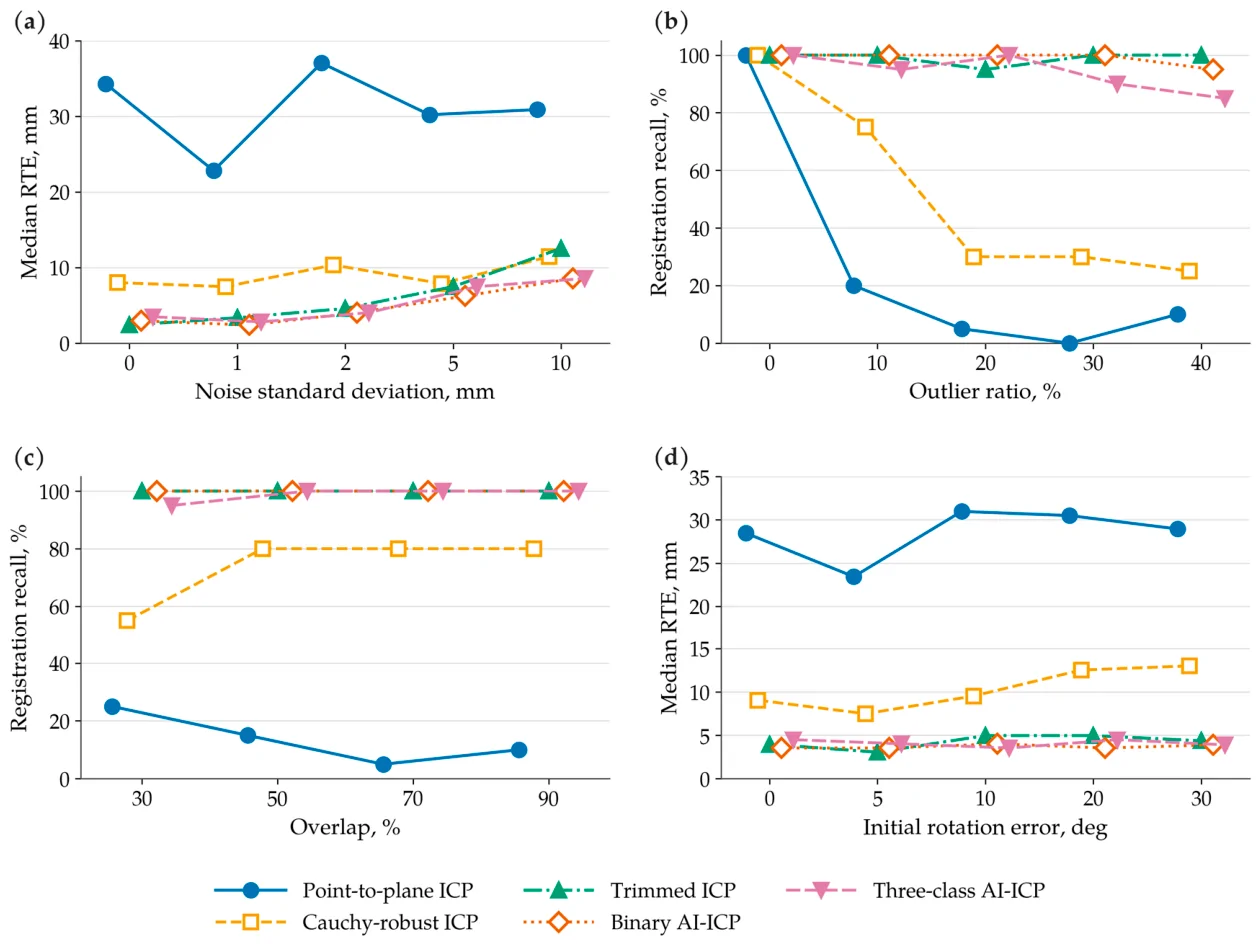
Sensitivity of registration error and recall to noise, outliers, overlap, and initial rotation; each point shows the median over 20 random trials. The legend labels “Binary AI-ICP” and “Three-class AI-ICP” denote the binary and three-class classifier-guided variants, respectively. Marker identity is also stated in the legend, so interpretation does not depend on colour alone. Panels: (**a**) noise, (**b**) outlier ratio, (**c**) overlap, (**d**) initial rotation. Each method has its own marker shape and line style; lines join the medians as a visual guide, and markers are offset slightly along the horizontal axis so that coincident values remain visible.

The benefit of learned reliability depended on the disturbance type. Under low noise and high overlap, trimmed ICP and both learned variants produced similar registration accuracy. As outlier contamination increased, the learned and trimmed methods delayed failure relative to untrimmed ICP. Low overlap and large initial rotation remained the most difficult conditions, demonstrating that correspondence weighting cannot replace adequate geometric support or coarse initialisation. Figure 4 shows the corresponding median sensitivity profiles.

### 6.3. Deformation Preservation

**Figure 5:**
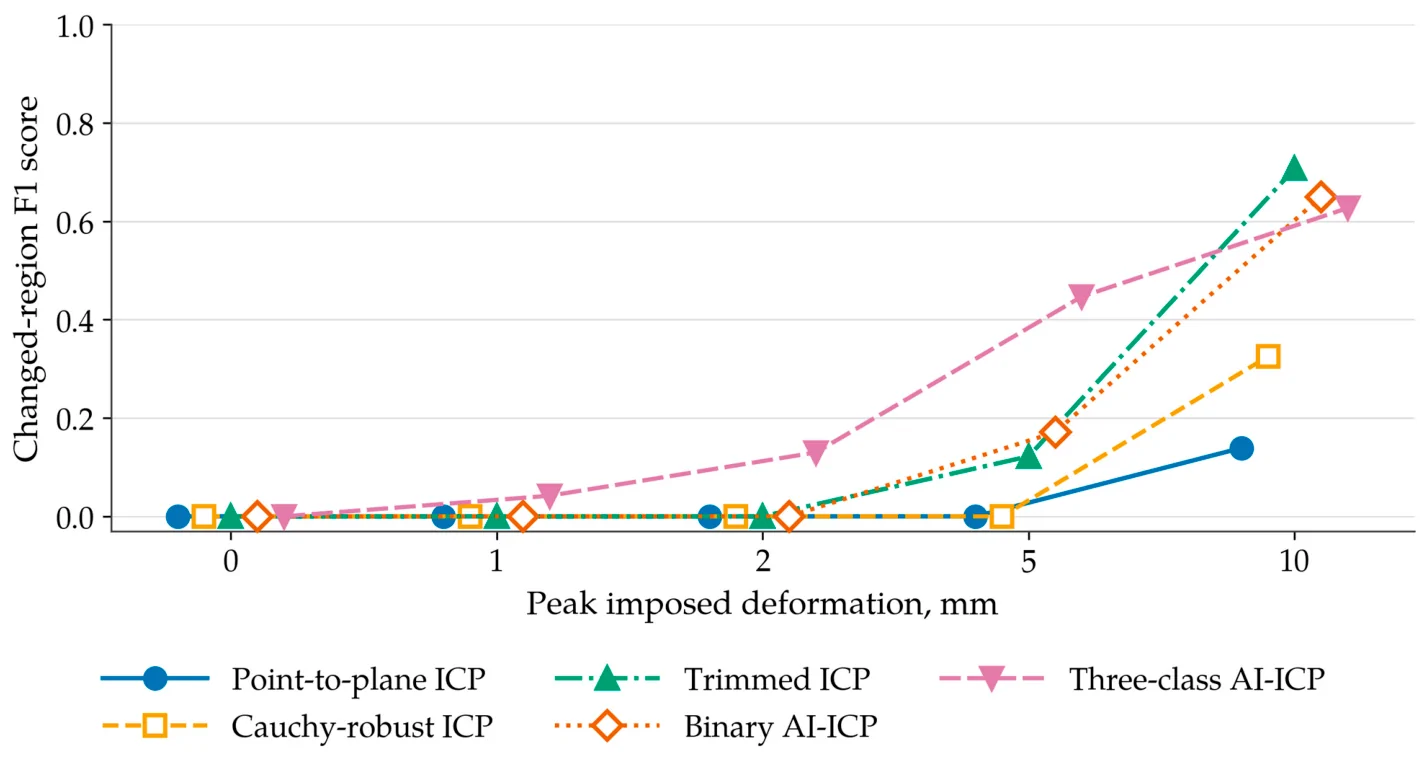
Changed-region F1 score versus imposed peak deformation. The explicit deformation class becomes most useful when the coherent displacement exceeds the noise scale. Marker shapes, line styles and the horizontal offset are as in Figure 4.

**Figure 6:**
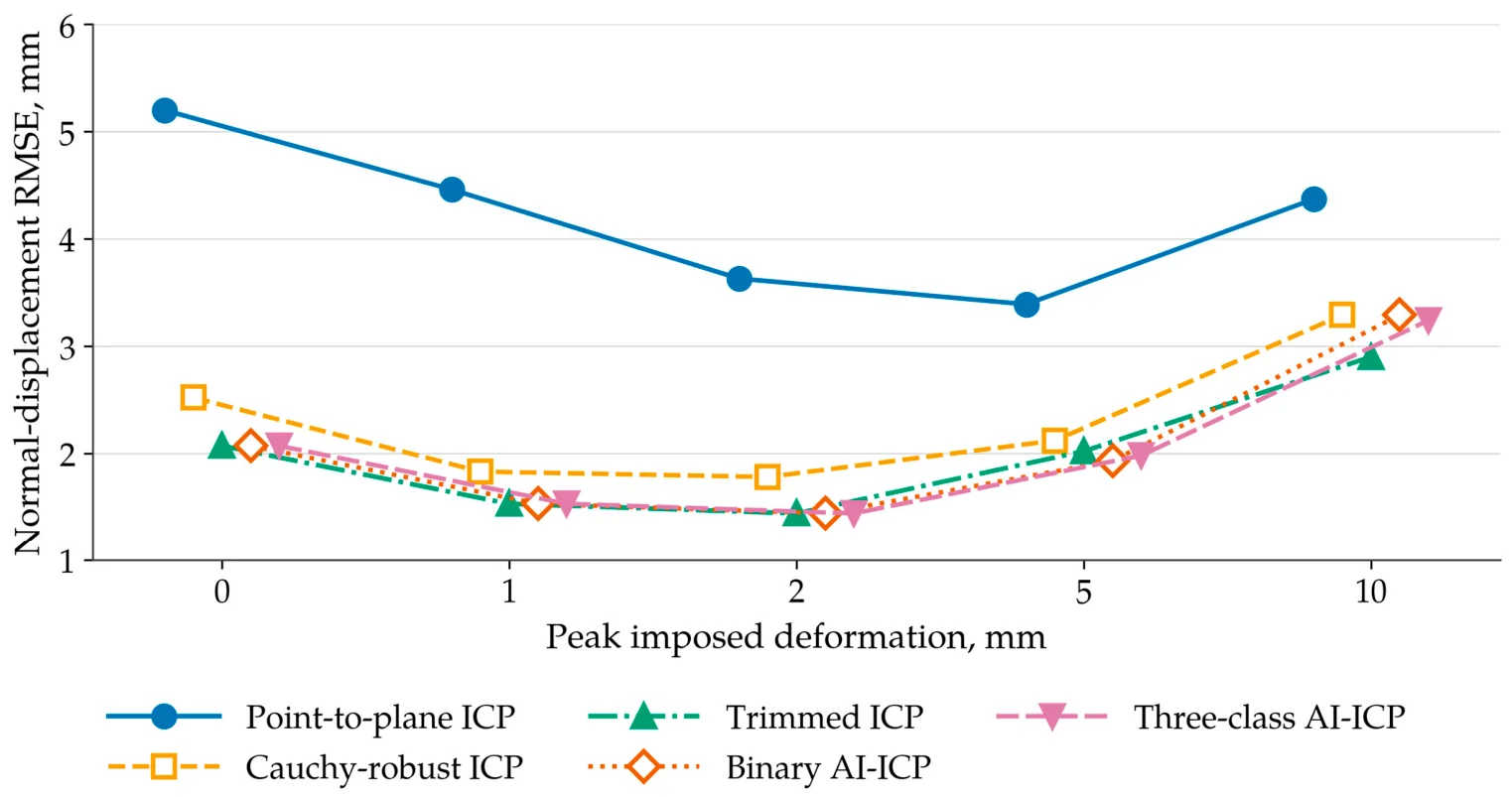
Normal-displacement RMSE versus imposed peak deformation for the compared registration variants. Marker shapes, line styles and the horizontal offset are as in Figure 4.

The deformation-preservation trade-off is summarised in Table 4. The explicit deformation class produced the highest changed-region F1 score of 0.524, compared with 0.413 for binary classifier-guided ICP and 0.221 for Cauchy-robust ICP. Its displacement RMSE of 2.585 mm was nearly identical to the binary-model value of 2.573 mm. The stable-region false-positive rate nevertheless increased to 6.71%, showing that improved deformation recall was obtained at the cost of additional false alarms. Figure 5 and Figure 6 respectively show how changed-region F1 and displacement RMSE vary with imposed peak deformation.

### 6.4. Ablation, Model Complexity, and Runtime

**Figure 7:**
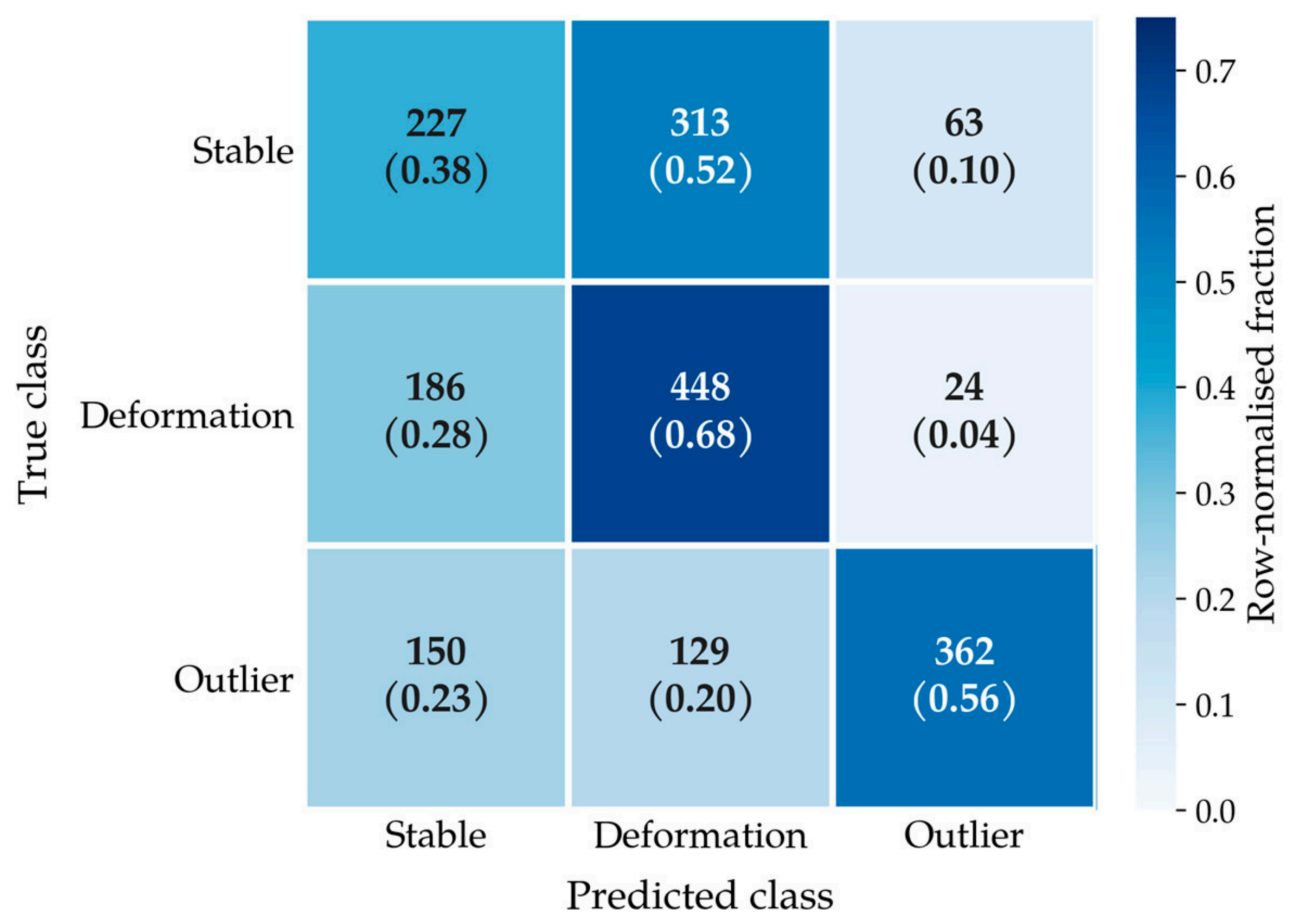
Confusion matrix of the selected compact three-class MLP on the internal synthetic test subset. Unseen-geometry performance is reported separately. Cells give the count and, in parentheses, the row-normalised fraction.

Table 5 summarises the ablation and runtime results. Soft reliability weighting was necessary for stable pose estimation. Replacing soft weights with a hard threshold reduced registration recall from 100.0% to 45.0%, despite a locally higher changed-region F1 than several simplified variants. Removing local residual features reduced F1 from 0.404 to 0.166, and removing the robust loss reduced it to 0.266. The full method therefore combined complementary learned and robust-geometric components rather than deriving its performance from one isolated element. The classwise error pattern of the selected MLP is shown in Figure 7. These ablations do not isolate correspondence-boundary errors; a dedicated boundary-distance analysis and a broader parameter/loss sweep remain necessary.

### 6.5. Generalisation and Failure Cases

On the held-out corner-frame geometry, the three-class MLP achieved macro-F1 = 0.413, compared with 0.449 for gradient boosting and 0.364 for logistic regression. The binary MLP reached macro-F1 = 0.756. The reduction relative to internal validation indicates a measurable geometry-domain shift and shows that synthetic training alone is not sufficient evidence of generalisation to real structures or sensors.

**Figure 8:**
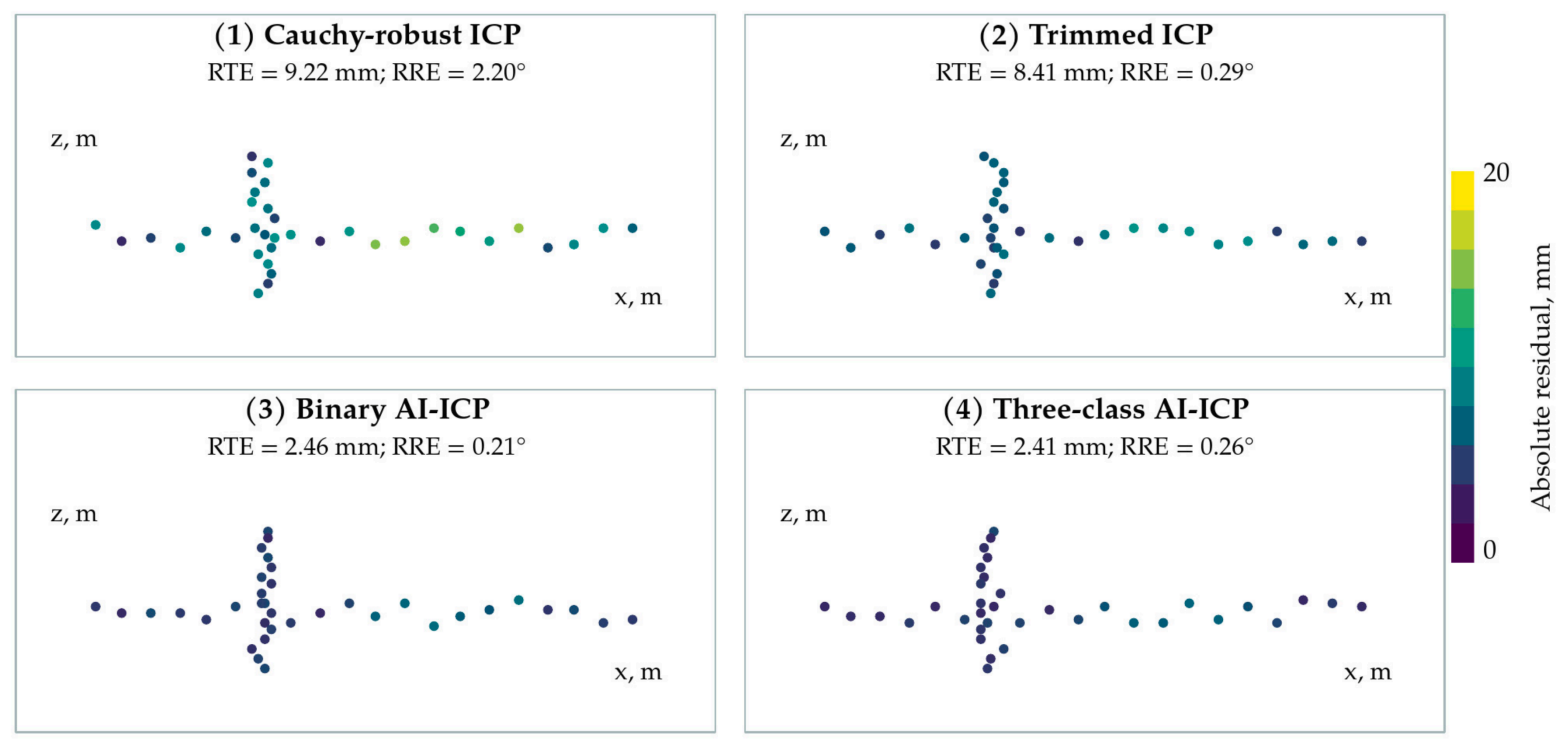
Qualitative residual maps for a held-out corner-frame pair: (**1**) Cauchy-robust ICP, (**2**) trimmed ICP, (**3**) binary classifier-guided ICP, and (**4**) three-class classifier-guided ICP. Conditions: 5 mm noise, 25% outliers, 55% overlap, 50% density retention, 15° initial rotation, and 8 mm peak deformation.

The principal synthetic failure cases were low overlap, large initial rotation, sparse moving clouds, and deformation occupying too much of the visible surface. In such cases, classification error, insufficient stable support, and geometric degeneracy must be distinguished. The algorithm should report support size and normal-matrix conditioning rather than return an apparently precise pose under weak observability. Representative held-out corner-frame residual maps are shown in Figure 8.

### 6.6. External Real-Scan Stress Test and Cross-Domain Transfer

The Stanford Bunny pair represents a clean, rigid, high-overlap external test rather than an engineering deformation case. The identity-start results are summarised in Table 6. Point-to-point ICP recovered the numerical reference most accurately, whereas the three-class model did not satisfy the success threshold.

**Figure 9:**
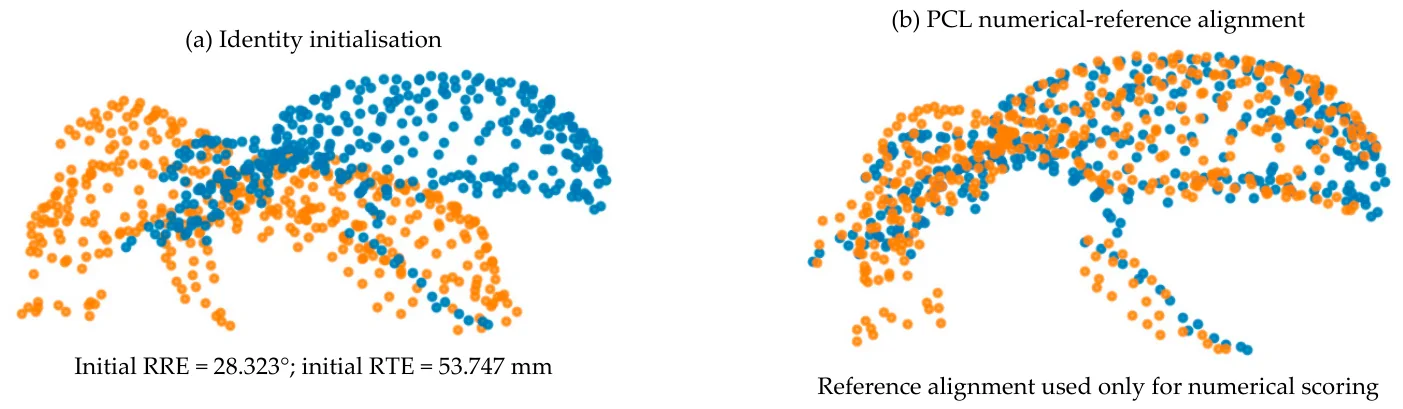
External Stanford Bunny scan pair before registration and after applying the PCL numerical reference transform. Blue and orange show the two independently acquired fragments. Stanford Bunny data: Stanford 3D Scanning Repository [48]; numerical reference transform: Point Cloud Library [46].

The initial misalignment and numerical-reference alignment are shown in Figure 9. Blue and orange denote the independently acquired source and target fragments. The figure is a rigid-registration visualisation; it contains no physical deformation signal.

The quantitative comparison of the six native-initialisation outputs is reported in Table 6, while Figure 9 provides the essential visual context for the external rigid-registration test. The learned weighting did not improve this clean, rigid, high-overlap case, for which ordinary geometric correspondences were already reliable.

Robustness over the 60 controlled starts is summarised in Table 7. Point-to-point ICP achieved a median RRE of 0.015° and median RTE of 0.020 mm, with 100% success. Point-to-plane ICP achieved 98.3% success. The Cauchy, trimmed, binary learned, and three-class learned variants did not satisfy the joint RRE/RTE criterion for any controlled start. Friedman tests detected method effects for both RTE and RRE (*p* < 10^−59^), and all Holm-adjusted pairwise comparisons involving the three-class method were significant.

Each of the 60 starts perturbs the reference pose by one of five rotation magnitudes (0°, 5°, 10°, 15°, 20°) and one of four translation magnitudes (0, 5, 10, 20 mm), with three random directions per combination, and every method is run from the same start. Success requires RRE < 2° and RTE < 5 mm at convergence. The two classical variants reach essentially the same solution from every start: point-to-point ICP gives a median RRE of 0.015° and a median RTE of 0.020 mm, and point-to-plane ICP 1.003° and 1.868 mm, with one of its 60 runs outside the threshold (98.3%). All four reweighted variants miss the threshold from every start, and this includes the two that involve no learning, Cauchy-robust ICP (2.534°, 2.502 mm) and trimmed ICP (6.597°, 4.068 mm). The three-class variant (4.911°, 3.821 mm) lies between these two, and the binary variant (5.844°, 4.153 mm) is close to trimmed ICP. The failure on this pair therefore cannot be attributed to the classifier alone: on a clean rigid pair of fewer than 400 points, downweighting or discarding correspondences removes constraints that the unweighted solution needs. The reweighted variants do not diverge. Their residual RMSE (4.04–4.60 mm) is close to that of point-to-point ICP (4.181 mm), and they stop after a median of 10.5–30 iterations at a pose a few degrees from the reference. A low residual is thus reached at a wrong pose, which is why residual RMSE is not accepted as evidence of correct registration (Section 5.6). Median runtime stays between 0.016 and 0.064 s per pair for all methods.

All methods used the same 60 controlled starts; Table 7 reports medians and success rates under the predefined criterion.

The cross-domain classifier scores explain the transfer failure. At the numerical reference pose, 397 correspondences were evaluated. The three-class MLP produced mean stable, deformation, and outlier scores of 0.370, 0.341, and 0.289, respectively; its hard-label split was 38.8% stable, 39.3% deformation, and 21.9% outlier. Because the Bunny pair is rigid, the 39.3% deformation label rate is a false cross-domain response rather than physical change. By contrast, the binary classifier produced a mean inlier score of 0.755. The result indicates that balanced synthetic training did not transfer directly to the real scan sampling, spacing, local surface statistics, and natural class prevalence.

Block B is therefore an explicit cross-domain failure analysis. It does not negate the controlled Block A result, but it prevents a claim that the Block A classifier transfers to real scan data without adaptation. Probability calibration, prior correction, limited real-domain tuning, or an out-of-distribution gate with a classical fallback should be evaluated before the scores are used operationally. One external pair cannot establish general external validity; broader evaluation on multiple real engineering scenes is required.

### 6.7. Public Benchmarks and Comparison with Stable-Area Methods and X-ICP

To provide a quantitative comparison, the proposed method, the five baselines of Section 5.5 and three further methods were run on public data. Because the comparison methods have no common open implementation, the whole pipeline was re-implemented in Python 3.14 (NumPy 2.5.4, SciPy 1.18.1 and scikit-learn 1.9.1) from the descriptions in Section 4. The three-class and binary classifiers were retrained on a re-implementation of the synthetic generator of Section 5.2. They reached an internal macro-F1 of 0.576 (0.544 in Section 5.3) and 0.499 on the held-out corner frame (0.413 in Section 6.5), and on the synthetic benchmark the re-implementation reproduced the ranking of Table 3 (binary and three-class ahead of trimmed and Cauchy-robust ICP, which are ahead of point-to-plane and point-to-point ICP) but not its absolute recall values. The figures in this section therefore come from a re-implementation and are not directly comparable with Table 3, Table 4, Table 5, Table 6 and Table 7. Parameters that Section 4 leaves open were fixed before the runs: d_max_ = 6 h, K = 60 iterations, γ = 1, a_min_ = 0.05, a Cauchy constant of 2.3849 and a trim ratio of 0.8.

X-ICP [36] was implemented with its published localisability thresholds (κ_f_ = cos 80°, a strong-contribution limit of cos 45°, κ_1_ = 250, κ_2_ = 180 and κ_3_ = 35) and with equality constraints on directions that are not localisable. The supervoxel-based stable-area method [43] and Piecewise-ICP [44] share a supervoxel segmentation in the spirit of voxel cloud connectivity segmentation [49], with a seed resolution of 6 h chosen from 4 h, 6 h and 8 h on eight pairs. The supervoxel method removes supervoxels whose mean normal distance exceeds a 95% confidence bound and refines the pose on the remaining points. Piecewise-ICP registers the centroids of planar patches to the target surface and removes patches with a shrinking distance threshold bounded below by the level of detection; its Kalman filtering applies to time series and was omitted. These are simplified versions written from the publications, not the original authors’ code.

Three datasets with reference poses were used. The first is augmented ICL-NUIM [50,51]: 59 pairs of depth frames, 20 frames apart, from the livingroom1 and office1 sequences, with simulated sensor noise, a voxel size of 5 cm and a median overlap of 0.82 (minimum 0.24). The second is 3DMatch [52]: six pairs of real fragments fused from colour-and-depth frames, with the official reference poses; two of them belong to the low-overlap 3DLoMatch split [28], and the overlap ranges from 0.23 to 0.61. The third consists of five scans of an indoor sequence of the ETH laser registration datasets [53], giving ten pairs with an overlap of 0.64–0.91 at a voxel size of 10 cm. Every pair was started from controlled perturbations of the reference pose, combining rotations of 0–20° with translations of 0–20 cm (ICL-NUIM and 3DMatch) or 0–1 m (ETH). This gave 1180, 360 and 200 runs per method. Success required RRE < 2° and RTE < 5 cm (10 cm for ETH). The classifiers were used without retraining unless stated otherwise.

Table 8 shows that no single method is best on all three datasets. Used without retraining, the three-class variant is in the leading group with Cauchy-robust, trimmed and binary classifier-guided ICP on every dataset. In paired Wilcoxon tests on RTE with Holm correction (Friedman *p* < 10^−200^ for each dataset), it was more accurate than point-to-plane ICP, X-ICP and the supervoxel method on all three datasets (*p* < 10^−11^), and its difference from Cauchy-robust ICP was not significant on ICL-NUIM and ETH. Piecewise-ICP had the highest success on ICL-NUIM (71.2% against 66.0%), where large planar patches are common, but it was weaker on the small 3DMatch fragments and from 1 m initial offsets on ETH. On ETH, point-to-plane ICP, Cauchy-robust ICP and X-ICP reached 98.0% success against 93.0% for the three-class variant, although the robust and learned variants had a median RTE near 3 mm against 20 mm for point-to-plane ICP and X-ICP. The 3DMatch figures rest on six pairs and are dominated by pair effects, so they are indicative only. The prior correction of Equation (23), applied inside the registration loop with the illustrative prior (0.90, 0.02, 0.08), did not change rigid accuracy measurably.

The deformation class was then tested on the same real-scene geometry. A Gaussian displacement field (Equation (21), s = 0.25 m) with a peak of 3, 6 or 10 cm was imposed on the moving frame of 20 ICL-NUIM pairs, with two starts each (5° and 5 cm). The changed region was the core defined by Equation (24) with τ = 0.5. Two detection rules were compared: a common residual threshold, |δ_i_| greater than three times 1.4826 MAD of all signed residuals, applied after each registration; and each method’s own change output, namely the deformation score of Equation (19) with D_i_ > 3 for the three-class variants and the removed supervoxels or patches for the two stable-area methods. Table 9 summarises the results.

Trained on synthetic data alone, the deformation class did not carry over: its D-score detected almost nothing (F1 = 0.065 at 10 cm). A label check at the reference pose showed why. Stable surface points received the deformation label in 34–63% of cases, and deformed points were labelled stable or unreliable, almost never deformation. Retraining with a wider synthetic amplitude range (0.3–4 times the point spacing instead of at most 0.36 times) removed most of the false deformation labels on stable surfaces but still did not recover the imposed change, so the limit was the synthetic-to-real gap and not only the amplitude range. A small real-domain calibration was therefore tested. The same MLP with the same 14 features was trained on 110 frame pairs of the livingroom2 and office2 sequences of the same benchmark, with Gaussian displacements of 1–4 times the point spacing, and was then applied without change to the livingroom1 and office1 pairs of Table 9.

The calibrated D-score reached F1 = 0.279 at 6 cm and 0.486 at 10 cm with a stable-region false-positive rate of 0.6%. Its F1 did not differ significantly from that of the best residual threshold (trimmed ICP; paired Wilcoxon *p* = 0.91 at 6 cm and *p* = 0.069 at 10 cm), but its false-positive rate was about ten times lower (*p* < 10^−7^). It was clearly better than the change output of Piecewise-ICP (*p* < 0.001), and the supervoxel method flagged more than half of the stable points. Under the 10 cm deformation the calibrated variant also had the highest registration success (70%). At 3 cm, close to the sensor noise, no method detected the change (F1 ≤ 0.11). Two limitations apply: the calibration and test sequences share the same two room models, with different camera paths, and the deformations are imposed numerically. The calibration also reduced robustness on 3DMatch at the loose threshold (76.4% against 95.8%, Table 8), which shows that it is specific to the sensor domain.

Finally, two real TLS epochs (2017 and 2018) of a subarea of the Äußeres Hochebenkar rock glacier [54] were used as a case in which real deformation covers the whole overlap. The reference was the stable-area transformation published with the data, which was estimated on stable terrain outside the subarea; inside the subarea the median nearest-neighbour distance between the epochs was about 0.3 m. Started at the reference pose, every method moved away from it, by 0.63 m for point-to-point ICP and by 2.2–2.5 m for all other methods, including both stable-area methods, because no stable ground was available inside the overlap. The three-class scores did signal the problem: only 2% of the correspondences were labelled stable (8% deformation and 90% unreliable). This supports the rule proposed in Section 6.5 that the stable support should be reported and a pose refused when that support is too small; the present implementation does not yet enforce such a rule.

## 7. Discussion

### 7.1. Registration and Change-Detection Trade-Off

The synthetic evidence supports a limited monitoring-oriented hypothesis. Learned reliability substantially improved registration relative to ordinary point-to-point, point-to-plane, and Cauchy-robust ICP. However, binary classifier-guided ICP was slightly better in median RTE and registration recall, and trimmed ICP was statistically close to the three-class method. The three-class formulation should therefore not be presented as universally superior for rigid pose estimation.

The monitoring-specific advantage appeared in changed-region discrimination. Explicitly representing coherent deformation increased F1 to 0.524, whereas binary classifier-guided ICP reached 0.413. A binary classifier is forced to treat changed points either as registration inliers, which can bias the pose, or as outliers, which can discard the signal. The third class avoids this forced choice by preserving candidate deformation after stable regions establish the rigid reference frame.

This improvement involved a trade-off. The three-class model produced a stable-region false-positive rate of 6.71%, higher than the 1.18% obtained by the binary model. Spatial connectivity, minimum-area constraints, and uncertainty-aware thresholds are therefore required before the residual map can support an engineering decision. Registration residuals alone must not be interpreted as proof of structural damage.

### 7.2. Model Complexity and Selection

The classifier results do not establish the MLP as the optimal model. Logistic regression and gradient boosting achieved higher internal macro-F1, while only the MLP was carried through the reported iterative ICP experiments. Its latency and model-size advantages justify it as an implementation candidate, but an end-to-end comparison of all three models is required before model selection can be treated as settled.

### 7.3. Limitations and Transfer Risks

Synthetic labels provide exact ground truth and controlled factor isolation, but they do not reproduce mixed pixels, reflectance effects, calibration drift, sensor-specific sampling, or natural class prevalence. Balancing the classes before training also changes the prior distribution. The softmax outputs are therefore useful as relative scores in Block A but cannot be assumed to be calibrated probabilities in a new sensor domain.

The external test also shows that learning is not automatically beneficial when classical geometry is well conditioned. On the clean, high-overlap Bunny pair, point-to-point ICP substantially outperformed the learned variants. A practical system should diagnose localisability and domain shift and permit a validated classical fallback instead of forcing classifier-derived weights in every case.

The evidence is further limited by one compact external pair, a software numerical reference rather than traceable ground truth, small synthetic point counts, and a one-factor-at-a-time design that does not quantify factor interactions. No physical deformation is present in Block B, and no direct experimental comparison with GICP, stable-area selection, or Piecewise-ICP was performed. The conclusions are therefore restricted to a controlled proof of concept and a documented transfer failure; metrological accuracy and field deployment remain unvalidated. The 50% synthetic deformation-core threshold and the classifier-to-pose weighting sharpness parameter were not subjected to dedicated sensitivity sweeps; such sweeps are required to establish parameter robustness. Boundary-distance analysis is also needed to separate boundary misclassification from possible over-regularisation by soft weighting.

### 7.4. Classwise Error Analysis: Where the Rigid-Accuracy Loss Comes from

The reviewers asked whether the lower rigid-registration accuracy of the three-class variant comes from over-regularisation by soft weighting or from misclassification. The confusion matrix in Figure 7 allows a partial answer without new experiments. Table 10 gives the per-class precision, recall and F1 computed from its counts; their unweighted mean reproduces the reported macro-F1 of 0.544.

The dominant error is confusion between the stable and deformation classes. Of the 603 stable test correspondences, 313 (51.9%) received a deformation hard label, and 186 of the 658 deformation correspondences (28.3%) a stable label. Together these two cells account for 499 of the 865 misclassifications (57.7%). If the stable and deformation classes are merged, 1174 of 1261 non-outlier correspondences (93.1%) are kept and the two-way accuracy rises from 54.5% to 80.8%. Outlier rejection is therefore not where the three-class model loses accuracy. The loss lies in separating stable from deforming geometry, which tends to lower the stable-class score, and hence the pose weight in Equation (10), on correspondences that are in fact stable. This agrees with both observations raised in review: the binary model, which does not have to make that separation, gives slightly better rigid accuracy (Table 3), and the three-class model raises more false alarms in stable regions (6.71% against 1.18%, Table 4).

Two qualifications apply. The hard-label confusion in Table 10 is larger than the 6.71% stable-region false-positive rate because the deformation map does not use hard labels: the score in Equation (19) multiplies the deformation-class score by a locally normalised residual and is followed by spatial filtering, so a deformation label on a point with a small residual does not produce an alarm. Additionally, the analysis identifies the class pair responsible but cannot say whether the confused correspondences lie near the edge of the deformation core, because distances to that edge were not recorded. That question needs the boundary-distance analysis listed in Section 7.3.

### 7.5. Class Prior Shift and a Prior Correction for Real Scans

Balanced training fixes the class prior at one third per class, whereas in a real monitoring scan deforming points are usually a small minority. Under such a label shift the class-conditional feature distributions are assumed unchanged and only the priors differ, in which case the balanced-prior scores can be converted to scores under a deployment prior by

(23) $${\tilde{\pi }}_{ij}^{c}=\frac{\pi_{ij}^{c}w_{c}}{\sum_{k\in \{S,D,O\}}\pi_{ij}^{k}w_{k}},w_{c}=\frac{p_{dep}\left(c\right)}{p_{train}\left(c\right)}$$

where $p_{train}\left(c\right)=1/3$ is the training prior and $p_{dep}\left(c\right)$ is the expected class prevalence in deployment. The corrected stable-class score then replaces $\pi_{ij}^{S}$ in Equation (10). Equation (23) needs no retraining; it needs only an estimate of the deployment prior, which can come from engineering judgement, from a small labelled sample, or from an iterative estimate on unlabelled scans.

As an illustration, applying Equation (23) to the mean Bunny score vector (0.370, 0.341, 0.289) with an assumed deployment prior of (0.90, 0.02, 0.08) for the stable, deformation and outlier classes gives (0.918, 0.019, 0.064). The deformation score drops from 0.341 to 0.019 and the stable score rises from 0.370 to 0.918. This shows the direction and size of the correction and nothing more: it was applied to a mean score vector, the prior is assumed and not estimated, and no registration run used corrected scores. Whether the correction restores registration success on the Bunny pair remains to be tested.

Prior shift is also not the whole explanation of the transfer failure. The mean Bunny score vector is close to uniform, with an entropy of 1.093 nats against a maximum of 1.099 (99.5%), so the classifier is close to uninformative on this pair and its hard labels split almost by chance. That points to covariate shift in the features. The median spacing of the Bunny fragments is 7.997 mm against 31.3 mm in the synthetic data, a factor of 3.9, and three of the fourteen features in Equation (7) are residuals in absolute millimetres that are not normalised by spacing. A prior correction rescales scores but cannot repair features that fall outside the training range, so it complements real-domain calibration and feature normalisation and does not replace them. It is also worth noting that the 39.3% deformation label rate on the rigid Bunny pair is not larger than the in-domain rate at which stable correspondences receive a deformation hard label (51.9%, Table 10). The hard-label rate alone is therefore a weak indicator of transfer; what fails on the Bunny pair is the pose weighting built on scores that no longer discriminate.

### 7.6. Deformation-Label Threshold and Weighting Sharpness: Analytical Sensitivity

Empirical sweeps of the deformation-label threshold and of the sharpness parameter γ were not run, as stated in Section 7.3. Both parameters can nevertheless be characterised analytically, which answers part of the question about their basis. For the Gaussian field of Equation (21), the set of points whose imposed displacement is at least a fraction τ of the peak amplitude *A* is the ellipse

(24) $${\left(\frac{x-c_{x}}{s_{x}}\right)}^{2}+{\left(\frac{y-c_{y}}{s_{y}}\right)}^{2}\leq 2ln\left(1/\tau \right)$$

For τ = 0.5 the right-hand side equals 2 ln 2 = 1.386, so the deformation core is the ellipse with semi-axes 1.177 *s*_x_ and 1.177 *s*_y_. This is the half-maximum contour of the field, whose width along each axis is the full width at half maximum (FWHM), 2.355 *s*. The 50% rule is therefore the standard convention for the extent of a Gaussian-shaped feature and was not tuned on the data. The area of the labelled core is 2π ln (1/τ) *s*_x_*s*_y_, that is 4.36 *s*_x_*s*_y_ at τ = 0.5. Lowering the threshold to 0.25 doubles this area (factor 2.00) and raising it to 0.75 reduces it to 0.42 of its value, so the number of deformation labels, and with it the class balance before resampling, is sensitive to τ in a known way. The displacement at the core boundary is τ*A*: 2.5 and 5 mm for the 5 and 10 mm cases, respectively, and 0.5 and 1 mm for the 1 and 2 mm cases, respectively, which is consistent with Table 4 treating only the 5 and 10 mm cases as detectable.

The sharpness parameter γ acts through Equation (10). Table 11 lists the pose weight for several stable-class scores and four values of γ, before clipping at *a*_min_. With γ < 1 the weights are flattened and the classifier has little influence; with γ = 1 the weight equals the score; with γ = 4 a correspondence with a stable score of 0.50 keeps about 6% of full weight and the scheme approaches a hard threshold. The ablation in Table 5 shows that a hard threshold reduced registration recall from 100.0% to 45.0%, so large values of γ are expected to be harmful for the same reason, while small values give up the benefit of the classifier. This bounds the useful range of γ from both sides but does not replace an empirical sweep, which remains to be done.

### 7.7. Relation to Stable-Area Methods, X-ICP and Global Registration

Table 12 sets the present formulation beside the methods that the reviewers asked to see compared. The table is qualitative. It records what each method decides and how that decision enters the pose estimate; it reports no accuracy figures, because none of these methods was run on the present data. Their accuracy on public benchmarks, obtained with simplified re-implementations, is reported in Section 6.7 and Table 8 and Table 9.

The stable-area methods and Piecewise-ICP decide at the level of segments, supervoxels or planar patches and then select the stable units outright. They need scans dense enough for the segmentation to be meaningful. The synthetic clouds used here hold about 700 points at a median spacing of 31.3 mm and were not designed for patch segmentation, so a fair numerical comparison needs a denser benchmark with patch-level ground truth; running these methods on the present data would mainly test the segmentation. X-ICP answers a different question, namely whether the retained constraints span all six degrees of freedom, and can be combined with any correspondence weighting, including the present one. FGR and FPFH with RANSAC provide the coarse pose and sit before the refinement stage. The proposed method is thus complementary to X-ICP and to the global methods and is a point-level alternative to the patch-level methods, and no claim is made that it outperforms any of them. A direct comparison on dense real scans is the first item of follow-up work.

## 8. Conclusions

This study introduced a deformation-preserving reliability-guided ICP formulation in which a compact three-class classifier assigns stable-anchor, deformation-candidate, and unreliable-match scores while the rigid pose remains determined by an explicit Cauchy-robust point-to-plane optimisation.

In the controlled synthetic benchmark, the three-class method achieved median RRE = 0.227°, median RTE = 4.103 mm, and registration recall = 96.61%. Its principal validated benefit was changed-region discrimination, where F1 reached 0.524, although binary classifier-guided ICP remained slightly better for rigid-registration accuracy and produced fewer stable-region false positives. In the external Stanford Bunny stress test, point-to-point ICP achieved 100% success over 60 starts, whereas the unretrained three-class method achieved 0% and assigned 39.3% of rigid-object correspondences to the deformation class. The combined evidence supports three-way correspondence modelling under controlled corruption but rejects any claim of calibration-free real-domain superiority.

A follow-up evaluation on public benchmarks, run with a re-implementation of the method and simplified re-implementations of X-ICP, a supervoxel-based stable-area method and Piecewise-ICP, placed the three-class variant among the robust methods for rigid registration but not ahead of them on every dataset. Trained on synthetic data alone, its deformation class did not detect changes in real geometry; after a small real-domain calibration it matched the best residual threshold in changed-region F1 at about one tenth of the false-positive rate. The prior correction of Equation (23) gave no benefit inside the registration loop. The study therefore remains diagnostic: it shows when the third class helps and what it needs, and it does not claim an operational monitoring tool.

Two further directions arise from the limitations discussed above. On the geometric side, the conditioning diagnostics of Section 4.4 rest on the spectrum of the weighted normal matrix and on how the correspondence set behaves from one iteration to the next. A formal treatment of these objects, drawing on recent work on induced mappings and sequential properties of function spaces [55] and on operator eigenvalue problems [56], could give the degeneracy check a firmer theoretical basis, and the recovery of a displacement field from signed residuals could be posed as an inverse problem with appropriate boundary conditions [57,58]. On the classifier side, the softmax scores could be complemented by rule-based decision logic expressed through logical equations [59] and by precedent-based classification with transparent decision rules [60], so that the assignment of a correspondence to the stable, deformation, or unreliable class becomes as auditable as the pose update itself.

## Figures and Tables

**Table 1:** Dataset and evaluation-role summary.

Dataset/ Specimen	Source/ Sensor	Point Count After Preprocessing	Nominal Spacing	Overlap	Deformation Ground Truth	Use
Synthetic engineering surfaces: ribbed panel, cylinder segment, box beam; held-out corner frame	Analytically generated from clean parametric surfaces	Reference 700–720; moving 98–800	31.3 mm median (29.5–35.0 mm)	30–90%	Exact imposed normal-displacement field, 0–10 mm peak	Training, validation, controlled test, and held-out-geometry test
Stanford Bunny PCL pair: bun0.pcd→ bun4.pcd	Cyberware 3030 MS range scans; compact PCL registration-test fragments	Source 397; target 361	7.997 mm	96.2% estimated symmetric overlap	PCL numerical reference rigid pose; not metrological ground truth	External real-scan registration and synthetic-to-real transfer test

**Table 2:** Controlled factor sweeps used in the synthetic evaluation. One factor was varied at a time; each factor level defines one block of 20 independently generated trials, and reference settings repeated across different sweeps remain separate blocks.

Controlled Factor	Level 1	Level 2	Level 3	Level 4	Level 5
Gaussian noise, mm	0	1	2	5	10
Outlier ratio, %	0	10	20	30	40
Overlap, %	30	50	70	90	—
Initial rotation, °	0	5	10	20	30
Retained density, %	100	50	25	12.5	—
Peak deformation, mm	0	1	2	5	10

**Table 3:** Registration performance over the complete controlled synthetic test set.

Method	Median RRE, °	Median RTE, mm	Residual RMSE, mm	Registration Recall, %	Numerical Failure Rate, %
Point-to-point ICP	1.823	9.200	32.605	43.04	0.0
Point-to-plane ICP	2.728	29.236	32.496	19.64	0.0
Trimmed ICP	0.209	4.186	32.419	94.64	0.0
Cauchy-robust ICP	0.729	9.069	32.346	66.96	0.0
Binary classifier-guided ICP	0.184	3.549	32.383	97.86	0.0
Proposed three-class classifier-guided ICP	0.227	4.103	32.299	96.61	0.0

**Table 4:** Deformation preservation and changed-region detection for the detectable 5 and 10 mm deformation cases.

Method	Displacement MAE, mm	Displacement RMSE, mm	Peak Bias, mm	Changed-Region F1	Stable-Region FPR, %
Point-to-plane ICP	4.635	5.531	6.670	0.135	11.37
Cauchy-robust ICP	2.305	2.730	0.228	0.221	4.69
Binary classifier-guided ICP	2.183	2.573	−0.208	0.413	1.18
Proposed three-class classifier-guided ICP	2.190	2.585	−0.240	0.524	6.71

**Table 5:** Ablation and computational cost on the dedicated synthetic ablation subset.

Variant	Registration Recall, %	Deformation RMSE, mm	Changed-Region F1	Runtime per Pair, s	Model Size, kB
Full three-class method	100.0	1.816	0.404	0.0314	94.0
Binary classifier	100.0	1.817	0.250	0.0224	93.0
Without learned weighting	85.0	2.183	0.065	0.0193	0.0
Hard reliability threshold	45.0	3.865	0.270	0.0212	94.0
Without local residual features	85.0	2.183	0.166	0.0231	94.0
Without robust loss	95.0	1.895	0.266	0.0266	94.0

**Table 6:** Registration performance from the native identity initialisation on the external real-scan pair.

Method	RRE, °	RTE, mm	Residual RMSE, mm	Iter.	Registration Success
Point-to-point ICP	0.001	0.004	4.181	20	Yes
Point-to-plane ICP	1.003	1.855	3.945	9	Yes
Cauchy-robust ICP	2.487	2.472	4.033	12	No
Trimmed ICP	6.548	3.903	4.584	14	No
Binary classifier-guided ICP	5.844	4.153	4.484	60	No
Three-class classifier-guided ICP	4.911	3.821	4.293	13	No

**Table 7:** Registration performance over 60 controlled initialisations on the external real-scan pair.

Method	Median RRE, °	Median RTE, mm	Median RMSE, mm	Registration Success, %	Median Iter.	Median Runtime, s
Point-to-point ICP	0.015	0.020	4.181	100.0	27	0.0471
Point-to-plane ICP	1.003	1.868	3.942	98.3	9	0.0158
Cauchy-robust ICP	2.534	2.502	4.040	0.0	10.5	0.0184
Trimmed ICP	6.597	4.068	4.602	0.0	15	0.0266
Binary classifier-guided ICP	5.844	4.153	4.484	0.0	30	0.0636
Three-class classifier-guided ICP	4.911	3.821	4.293	0.0	12	0.0248

**Table 8:** Registration on public benchmarks with the re-implemented methods: success rate and median translation error. Methods marked * are simplified re-implementations of [36,43,44]. Success requires RRE < 2° and RTE < 5 cm (ICL-NUIM, 3DMatch) or 10 cm (ETH); the 3DMatch column at 5°/10 cm uses a looser threshold. The calibrated three-class model is described in the text below Table 9.

Method	ICL-NUIM Success, %	ICL-NUIM Median RTE, cm	3DMatch Success, %	3DMatch Success at 5°/10 cm, %	ETH Success, %	ETH Median RTE, mm
Point-to-point ICP	29.7	8.30	0.0	16.7	97.5	34.8
Point-to-plane ICP	60.6	4.08	14.4	97.5	98.0	19.9
Trimmed ICP	62.3	3.01	43.3	75.3	94.5	2.7
Cauchy-robust ICP	67.8	3.05	31.1	90.3	98.0	2.8
Binary classifier-guided ICP	63.6	2.80	47.8	79.4	86.5	2.9
Three-class classifier-guided ICP	66.0	2.78	46.9	95.8	93.0	3.0
Three-class with prior correction, Equation (23)	65.5	2.87	45.8	93.1	89.5	2.8
Three-class, real-domain calibrated	65.2	2.88	45.8	76.4	95.0	2.8
X-ICP *	62.5	4.08	14.4	86.7	98.0	19.9
Supervoxel stable areas *	56.9	4.42	14.4	81.4	79.0	51.2
Piecewise-ICP *	71.2	2.95	18.6	55.3	75.5	3.6

**Table 9:** Changed-region detection on ICL-NUIM geometry with an imposed Gaussian deformation (20 pairs, two starts each; means). The stable-region false-positive rate (FPR) is measured on points with less than 5% of the peak displacement; success uses RRE < 2° and RTE < 5 cm at the 10 cm amplitude. Methods marked * are simplified re-implementations.

Method and Detection Rule	F1, 6 cm	F1, 10 cm	Stable FPR at 6 cm, %	Stable FPR at 10 cm, %	Success at 10 cm, %
Three-class, real-domain calibrated: D-score, Equation (19)	0.279	0.486	0.6	0.6	70.0
Three-class, real-domain calibrated: residual threshold	0.281	0.433	6.4	6.0	70.0
Three-class, synthetic training: D-score, Equation (19)	0.056	0.065	0.8	0.8	60.0
Three-class, synthetic training: residual threshold	0.212	0.365	5.9	5.5	60.0
Three-class with prior correction: D-score, Equation (19)	0.000	0.000	0.0	0.0	60.0
Binary classifier-guided ICP: residual threshold	0.239	0.395	6.2	5.8	60.0
Trimmed ICP: residual threshold	0.270	0.428	6.7	6.2	62.5
Cauchy-robust ICP: residual threshold	0.210	0.376	5.6	5.3	60.0
X-ICP *: residual threshold	0.146	0.287	4.6	4.2	30.0
Supervoxel stable areas *: removed supervoxels	0.119	0.127	59.5	56.6	45.0
Piecewise-ICP *: removed patches	0.192	0.247	7.8	7.2	65.0

**Table 10:** Per-class precision, recall and F1 of the three-class MLP on the internal synthetic test subset (1902 correspondences), computed from the confusion matrix in Figure 7.

Class	True Count	Predicted Count	Precision	Recall	F1
Stable	603	563	0.403	0.376	0.389
Deformation	658	890	0.503	0.681	0.579
Outlier	641	449	0.806	0.565	0.664
Macro average/accuracy	1902	1902	0.571	0.541	0.544/0.545

The last row gives the macro-averaged precision, recall and F1, followed by the overall accuracy.

**Table 11:** Pose weight (π^S^)^γ^ of Equation (10) for selected stable-class scores and sharpness values, before clipping at *a*_min_. The last row gives the ratio between the weights of a confident (0.90) and an ambiguous (0.50) correspondence.

Stable-Class Score	γ = 0.5	γ = 1	γ = 2	γ = 4
0.90	0.949	0.900	0.810	0.656
0.70	0.837	0.700	0.490	0.240
0.50	0.707	0.500	0.250	0.063
0.37 (Bunny mean)	0.608	0.370	0.137	0.019
Weight ratio, 0.90 vs. 0.50	1.34	1.80	3.24	10.50

**Table 12:** Qualitative positioning of the proposed formulation against stable-area methods, X-ICP and global registration. No accuracy comparison is implied.

Method	Decision Unit	What Is Decided	Use in Pose Estimation	Separate Class for Coherent Change	Run on the Present Data
Stable-area identification [42]	Segment	Stable or deformed segment	Hard selection of stable segments	No	No
Supervoxel-based stable areas [43]	Supervoxel	Stable or deformed supervoxel	Hard selection of stable supervoxels	No	No
Piecewise-ICP [44]	Planar patch	Stable or changed patch, with a level-of-detection threshold	Hard selection of stable patches	No	No
X-ICP [36]	Correspondence and pose direction	Localisability of each pose direction	Constrains the update in weak directions	Not addressed	No
FGR [18]; FPFH + RANSAC [19,20]	Feature correspondence	Inlier or outlier match	Global coarse pose	No	Coarse stage only
This work	Point correspondence	Stable, deformation candidate, or unreliable	Soft weight from the stable-class score	Yes	—

## Data Availability

The data presented in this study are openly available in The Stanford Bunny data at https://graphics.stanford.edu/data/3Dscanrep (accessed on 17 July 2026), reference [46,48]. The synthetic-generation code, configuration files, trained weights, and evaluation outputs are available from the corresponding author upon reasonable request.

## Abbreviations

The following abbreviations are used in this manuscript:

FGR	Fast global registration
FPFH	Fast point feature histogram
FPR	False-positive rate
FWHM	Full width at half maximum
GICP	Generalized iterative closest point
ICP	Iterative closest point
IRLS	Iteratively reweighted least squares
MAD	Median absolute deviation
MAE	Mean absolute error
M3C2	Multiscale model-to-model cloud comparison
MLP	Multilayer perceptron
PCL	Point Cloud Library
RANSAC	Random sample consensus
ReLU	Rectified linear unit
RMSE	Root-mean-square error
RRE	Relative rotation error
RTE	Relative translation error
SE(3)	Special Euclidean group of rigid transformations in three dimensions
SO(3)	Special orthogonal group of rotations in three dimensions
TLS	Terrestrial laser scanning
